# Natural Guardians: Natural Compounds as Radioprotectors in Cancer Therapy

**DOI:** 10.3390/ijms25136937

**Published:** 2024-06-25

**Authors:** Anna Stasiłowicz-Krzemień, Anna Gościniak, Dorota Formanowicz, Judyta Cielecka-Piontek

**Affiliations:** 1 Department of Pharmacognosy and Biomaterials, Poznan University of Medical Sciences, Rokietnicka 3, 60-806 Poznan, Poland; astasilowicz@ump.edu.pl (A.S.-K.); agosciniak@ump.edu.pl (A.G.); 2 Department of Medical Chemistry and Laboratory Medicine, Poznan University of Medical Sciences, Rokietnicka 8, 60-806 Poznan, Poland; doforman@ump.edu.pl

**Keywords:** plant materials, cancer, photoprotection, radiotherapy

## Abstract

Cancer remains a significant global health challenge, with millions of deaths attributed to it annually. Radiotherapy, a cornerstone in cancer treatment, aims to destroy cancer cells while minimizing harm to healthy tissues. However, the harmful effects of irradiation on normal cells present a formidable obstacle. To mitigate these effects, researchers have explored using radioprotectors and mitigators, including natural compounds derived from secondary plant metabolites. This review outlines the diverse classes of natural compounds, elucidating their roles as protectants of healthy cells. Furthermore, the review highlights the potential of these compounds as radioprotective agents capable of enhancing the body’s resilience to radiation therapy. By integrating natural radioprotectors into cancer treatment regimens, clinicians may improve therapeutic outcomes while minimizing the adverse effects on healthy tissues. Ongoing research in this area holds promise for developing complementary strategies to optimize radiotherapy efficacy and enhance patient quality of life.

## 1. Introduction

Cancer is believed to be a group of diseases that can occur anywhere in the body, and involves uncontrolled, abnormal cell growth that spreads or invades other parts of the organism. In 2022, global cancer statistics from the International Agency for Research on Cancer revealed close to 20 million new cases of cancer and 9.7 million deaths from cancer [1]. The data suggest that approximately one in five men or women will develop cancer in their lifetime, while around one in nine men and one in 12 women will die from it. According to US National Cancer Institute, it is projected that 2,001,140 new cancer cases will be identified in the United States in 2024, and 611,720 individuals will die from the disease [2]. Most types of cancer seem to evolve from one cell, which is genetically damaged and divides uncontrollably.

Irradiation might be used as a radical stand-alone cancer treatment or as a part of a combination therapy that includes surgeries, chemotherapy or/and hormonal therapy, immunotherapy, and hyperthermia [3,4,5,6]. Radiotherapy might be performed as an add-on before surgery to reduce the size of a tumor or after the procedure to radiate the surgical site to relegate the residual cancer cells. Irradiation likewise plays a role in palliative care [7]. X-rays, and alpha, beta, and gamma ionizing radiation (IR) are exploited in therapy [8]. A free radical, an atom or molecule carrying in its outer shell one or more unpaired electrons, is capable of independent existence, damages DNA, and causes the death of healthy and cancer cells [9].

According to Cox et al. [10], there are less sensitive tissues (e.g., the bile ducts, uterus, and vagina) that can tolerate an approximately 100-times higher doses than sensitive tissues (e.g., bone marrow, lens, and gonads). Post-radiation effects occur after hours or days on acutely responding tissues, like arterioles, bladder, bone marrow, capillaries, colon, esophagus, larynx, lymph node, oral mucosa, ovary, salivary gland, skin, small bowel, stomach, testis, and vagina. Tissues that reveal radiation effects in a few weeks to a few months after radiotherapy are categorized as sub-acutely responding tissues: brain, heart, kidney, liver, lung, and spinal cord. Late effects can occur after sufficient doses of radiation in all tissues. Nevertheless, several tissues manifest only late effects without previous acute effects: bile ducts, bone, breast, cartilage, lymph vessels, pancreas (endocrine), pituitary, thyroid, and uterus.

**Figure 1:**
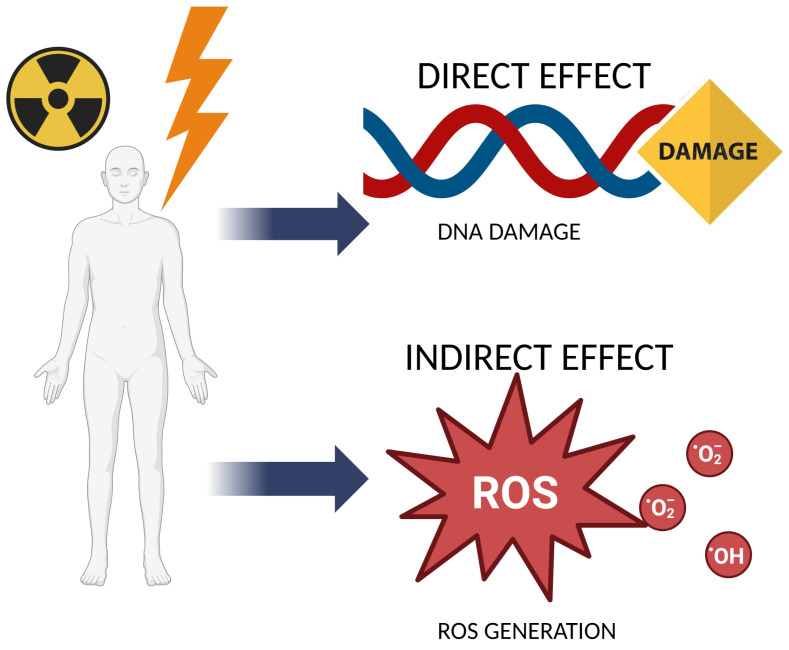
The mechanisms of the harmful effects of ionizing radiation (Abbrev.—reactive oxygen species (ROS)).

The mechanism of the harmful effects of ionizing radiation (IR) is strongly related to the increase in oxidative stress in irradiated tissues (Figure 1). It is known that IR can penetrate the cells of living organisms, causing the ionization of organic and inorganic compounds. Due to the high water content in cells, infrared radiolysis of water molecules is the primary process contributing to the increased formation of reactive oxygen species (ROS) and oxidative stress. ROS can also occur because mitochondria are IR-susceptible, and their dysfunction increases ROS generation. Next, ROS react rapidly with macromolecules, leading to cell dysfunction and apoptotic death.

Moreover, by altering DNA or proteins responsible for stabilizing the DNA structure, IR can also indirectly affect oxidative–antioxidant homeostasis in the cells, causing affected cells to become more susceptible to ROS damage (Figure 1). Besides, antioxidants or genes encoding enzymatic antioxidants may be damaged by IR, increasing oxidative stress [11]. Einor et al. [12], in a meta-analysis based on 41 studies on various biological matrices, showed that IR generates ROS even at low doses. Moreover, in studies on both in vivo and in vitro models, it was noted that irradiation increased the concentration of malondialdehyde (MDA), nitroxyl anion (NO•), and calcium ions and decreased superoxide dismutase (SOD) and catalase (CAT) activity and glutathione (GSH) concentration [11,13,14]. Szumiel [15] has proposed a relation between IR-induced oxidative stress, changes in mitochondrial DNA, and epigenetic changes in nuclear DNA, where IR-induced ROS generation causes mutations in mitochondrial DNA, which in turn contributes to ROS generation, creating a vicious circle. Mitochondrial DNA mutations affect the nuclear DNA epigenetic control mechanisms; they reduce methyltransferase activity, thus causing global DNA hypomethylation [16]. Hence, direct IR-related adverse side effects may develop as a result of increased oxidative stress, and ROS-related diseases may also develop. Therefore, it is crucial to identify effective and safe preventive compounds that protect human health tissues against IR damage [17].

On the other hand, it should be noted that ROS have a dual role in cancer development. They may lead to epigenetic alterations that promote the acceleration of tumor progression. Oxidative stress can induce nuclear DNA activation, which leads to the initiation of cancer. Moreover, ROS also inhibit T cells and natural killer (NK) cells and promote M2 macrophage recruitment and macrophage polarization; consequently, cancer cells can, in this way, escape immune surveillance and immune defenses. Moreover, ROS promote tumor invasion and metastasis by inducing epithelial–mesenchymal transition in cancer cells.

On the other hand, massive ROS accumulation can lead to tumor growth inhibition in two ways. Firstly, it blocks tumor cell proliferation by inhibiting the proliferation, cell cycle, and nucleotide and adenosine 5γ-triphosphate (ATP) biosynthesis signaling pathway. Secondly, it induces tumor cell death via activation of stress in the endoplasmic reticulum, apoptosis in mitochondrial and p53 pathways, and the ferroptosis pathway. However, cancer cells can adapt to ROS through a self-adaptation system, while healthy cells cannot. By contrast, on the other side, higher levels of ROS promote genome instability, inducing activation of cancer cell death or inhibiting resistance to anticancer treatment [18].

All this shows how complex oxidative stress is in the context of cancer and healthy cells. Radiotherapy, which impacts ROS generation, aims to destroy cancer cells and, at the same time, save as many normal cells as possible [19]. Regrettably, nowadays, there is no technique to exclude the degenerative consequences of irradiation entirely on normal cells; thus, it is crucial to come up with possibilities that can forcefully neutralize its side effects. Radiosensitizers increase the sensitivity of tumor cells to treatment; the cells become more vulnerable to the treatment after being damaged by the agent, or the damage will escalate with the irradiation [20].

Substances used to protect normal tissue cells depending on the administration time, with respect to radiation exposure time, can be divided into radioprotectors, mitigators, and treatment [21]. Radioprotectors are agents delivered before or at the time of irradiation to minimize the damage in healthy cells. Mitigators might be administered even after radiation exposure. Treatment is used after radiotherapy to expiate the normal tissue injury and improve the quality of life.

Radioprotectors and mitigators can be derived from secondary plant metabolites found in various plants. These natural compounds play a role in enhancing the body’s resilience to radiation therapy. Research in this area is ongoing, and identifying specific plant-derived compounds with radioprotective or mitigative effects holds promise for developing complementary strategies to enhance the effectiveness of radiotherapy while minimizing damage to healthy cells. Integrating these natural compounds into cancer treatment approaches could improve outcomes and reduce side effects for individuals undergoing radiation therapy.

Secondary plant metabolites are a diverse group of organic compounds produced by plants that go beyond the essential functions of growth, development, and reproduction. Unlike primary metabolites, which are critical to the basic life processes of plants, secondary metabolites serve various ecological roles, primarily in interactions with the environment, defense mechanisms, and adaptation to stressors [22].

The terpenes class encompasses monoterpenes like limonene and pinene, sesquiterpenes (caryophyllene and farnesene), diterpenes such as taxol and gibberellins, and complex polyterpenes like gutta-percha [23,24,25]. Phenolics, including coumarins (umbelliferone and aesculin), furanocoumarins (angelicin and bergapten), stilbenes (resveratrol and pterostilbene), flavonoids (kaempferol and hesperidin), and tannins (ellagic acid and catechins), play vital roles in plant defense and antioxidant activity [26,27,28]. Nitrogen-containing compounds comprise alkaloids (morphine and emetine), cyanogenic glucosides (amygdalin and prunasin), and non-protein amino acids (canavanine and mimosine) [29,30,31]. The sulfur-containing compounds class contains glucosinolates (sulforaphane), phytoalexins (camalexin), phytochelatins, and thionins (viscotoxin) [32,33,34,35]. Many isolated substances, plant extracts, and powders have been researched to be radioprotective agents.

## 2. Mechanisms of Radioprotective Effects

**Figure 2:**
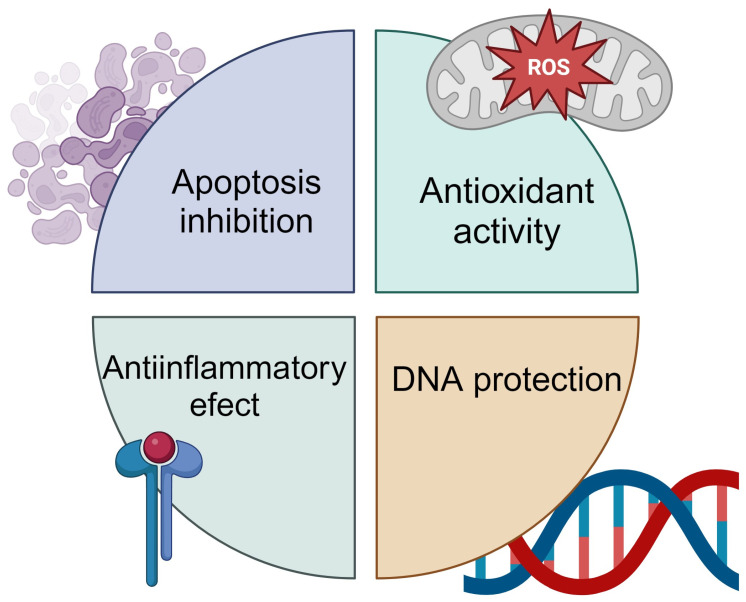
Mechanisms of radioprotective effects of plant compounds. (Abbrev.—reactive oxygen species (ROS)).

Radioprotective effects of plant substances have been attributed to various mechanisms, including immunostimulatory activity, antioxidant properties, DNA repair modulation, and an impact on apoptosis (Figure 2). In contrast to synthetic radioprotectors like thiol, plant compounds offer a safer and more economical option, being less toxic and often derived from substances commonly used in traditional medicine. As pointed out by Dowlath et al. [36], for a compound to be an ideal radioprotector, several functional properties must be present: (a) repair DNA and cell damage; (b) help rebuild damaged organs; (c) immunomodulation; (d) scavenging free radicals; and (e) oxidative stress reduction. These plant compounds meet the criteria for ideal radioprotectors due to their various mechanisms of action that effectively shield against the harmful effects of radiation [37]. Additionally, many plant-origin substances boast extended half-lives and show minimal interactions with other drugs administered during therapy. Similarly, terpenes have been implicated in radioprotective effects by upregulating nuclear factor erythroid 2-related factor 2 (Nrf2). This master transcription factor regulates antioxidant responses, thereby protecting cells from radiation-induced oxidative stress [38].

### 2.1. Antioxidant Activity

**Figure 3:**
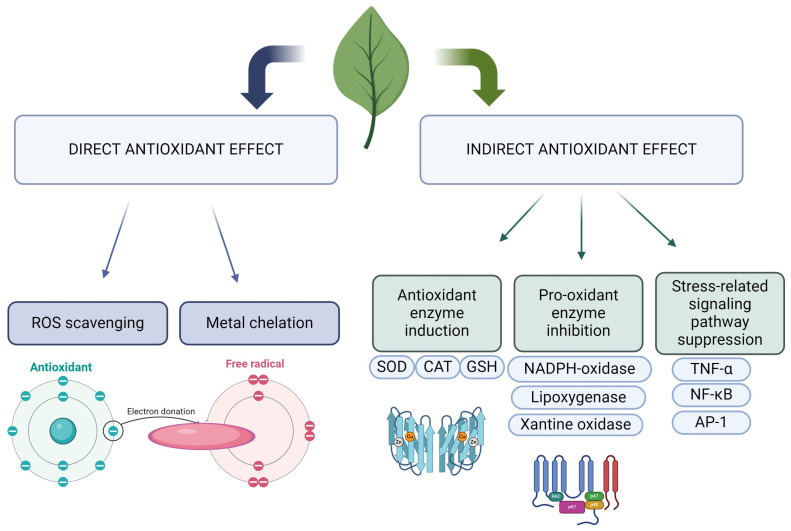
Antioxidant mechanisms of natural compounds (Abbrev.—reactive oxygen species (ROS), superoxide dismutase (SOD), catalase (CAT), glutathione (GSH), nicotinamide adenine dinucleotide phosphate oxidase (NADPH-oxidase), tumor necrosis factor (TNF), nuclear factor kappa-light-chain-enhancer of activated B cells (NF-κB), and activator protein 1 (AP-1)).

Natural substances can exhibit protective effects on cells exposed to radiation due to their potent antioxidant activity. The antioxidant mechanisms include both direct actions, such as free radical scavenging and metal chelation, and indirect mechanisms (Figure 3). Indirect mechanisms involve the induction of antioxidant enzymes such as superoxide dismutase (SOD), catalase (CAT), and glutathione (GSH), as well as the inhibition of pro-oxidant enzymes like NADPH oxidase, lipoxygenase, and xanthine oxidase [39]. These actions help to reduce oxidative stress in cells. The transfer of electrons to free radicals by phenolic compounds interrupts oxidative reactions, thereby mitigating the harmful effects of ROS generated by radiation [40]. Compounds such as polyphenols, which possess some of the strongest antioxidant properties, also affect pathways closely associated with oxidative stress, such as TNF-alpha and NF-kappa B [41]. Through these multifaceted mechanisms, natural substances can effectively mitigate the oxidative damage caused by radiation. Specifically, flavonoids have been reported to scavenge free radicals, inhibit cell apoptosis, and promote DNA repair, thereby mitigating radiation-induced damage [42,43].

### 2.2. DNA Protection

**Figure 4:**
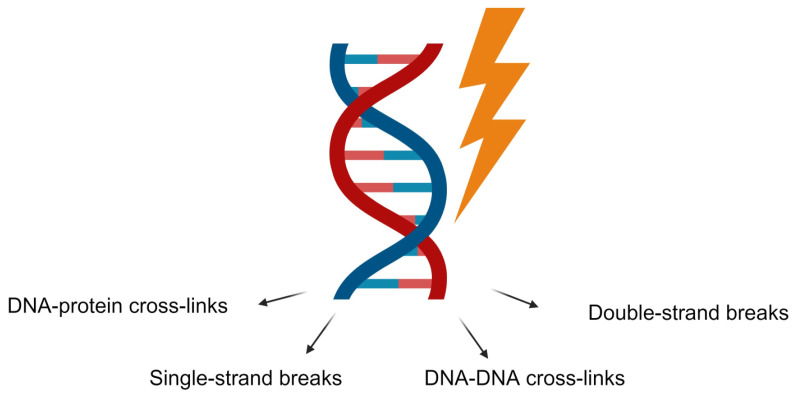
DNA damage induced by radiation.

Radiation can directly lead to DNA damage, such as single- or double-strand breaks (DSBs), base damage, and DNA–DNA or DNA–protein cross-links, with these changes clustering as complex, localized, multiple damaged sites (Figure 4) [44]. DNA-DSB damage is considered the most deadly event following ionizing radiation [45]. Radioprotectants can regulate DNA repair genes and protect against radiation-induced damage by ensuring the repair of DNA lesions [46]. This regulation helps mitigate the effects of ionizing radiation and maintain genomic stability [47]. Radioprotectants help mitigate radiation-induced damage by upregulating DNA repair genes and enhancing DNA repair pathways like homologous recombination (HR) and non-homologous end joining (NHEJ). They also boost base excision repair (BER) and nucleotide excision repair (NER) mechanisms, ensuring efficient repair of oxidative lesions [41,48,49]. Additionally, radioprotectants increase the activity of antioxidant proteins, reducing oxidative damage and the burden on DNA repair systems [50].

### 2.3. Apoptosis Inhibition

**Figure 5:**
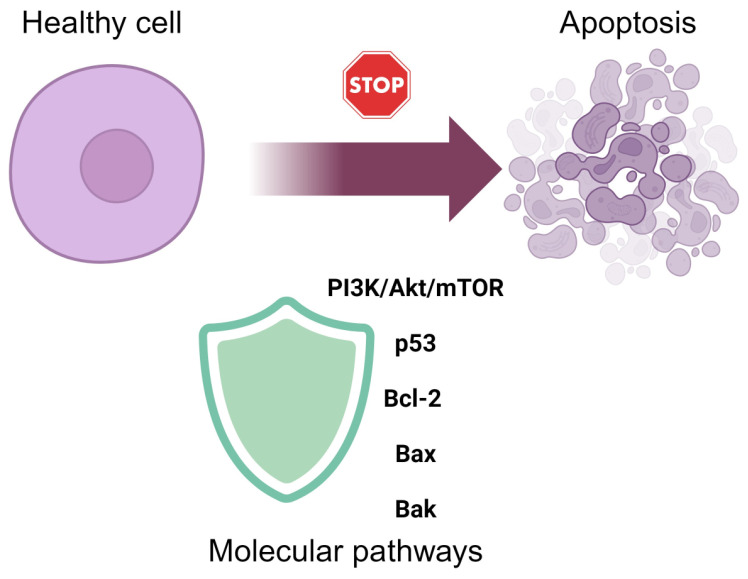
Mechanisms of inhibiting apoptosis.

Apoptosis, or programmed cell death, is a crucial process that allows the body to remove damaged or unwanted cells [51]. In the context of radiation exposure, healthy cells can undergo apoptosis due to the oxidative stress and DNA damage induced by radiation. Natural radioprotectors can play a significant role in preventing apoptosis in these cells, thereby mitigating the harmful effects of radiation [52]. Natural radioprotectors can influence several key molecular pathways involved in apoptosis (Figure 5). One such pathway is the PI3K/Akt/mTOR pathway, which promotes cell survival and growth. Activation of this pathway by natural compounds can enhance cell survival by inhibiting pro-apoptotic signals [53]. Additionally, the p53 protein, known as the “guardian of the genome”, is a critical regulator of apoptosis in response to DNA damage [54]. Natural radioprotectors can inhibit the activity of p53, thereby reducing the initiation of the apoptotic process [55]. Such activity concerns, among others, apigenin [56]. Furthermore, the Bcl-2 family of proteins, including Bcl-2, Bax, and Bak, plays a pivotal role in the regulation of apoptosis. Bcl-2 is an antiapoptotic protein that promotes cell survival, while Bax and Bak are pro-apoptotic proteins that promote cell death [57]. Natural radioprotectors can increase the expression of Bcl-2 while decreasing the levels of Bax and Bak, thus tipping the balance towards cell survival rather than apoptosis [57]. The PI3K/AKT/mTOR signaling pathway has been implicated in the apoptotic effects of certain plant extracts on cancer cells [58].

### 2.4. Antiinflammatory Effect

**Figure 6:**
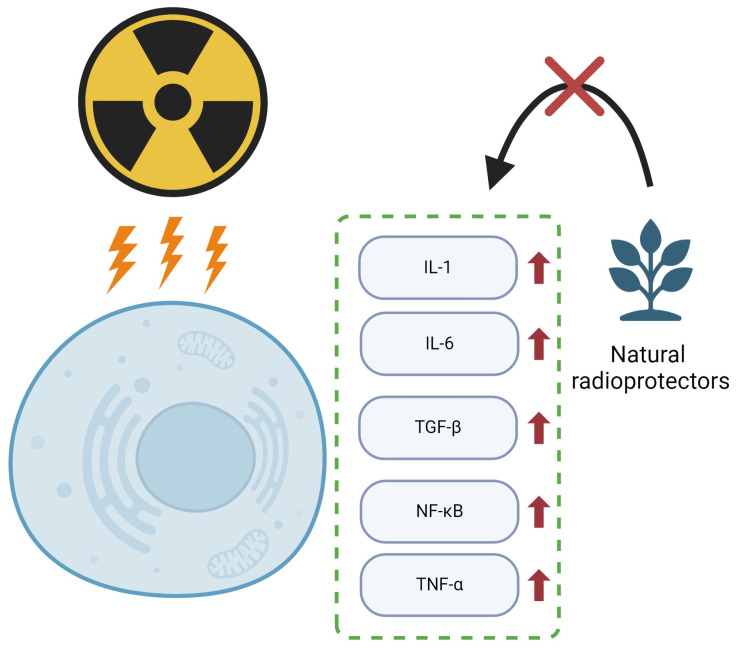
Mechanisms of natural radioprotectors in reducing radiation-induced inflammation (Abbrev.—interleukin (IL), transforming growth factor β (TGF-β), nuclear factor kappa-light-chain-enhancer of activated B cells (NF-κB), and tumor necrosis factor α (TNF-α)).

Radiation therapy can lead to extensive damage to the body, including the activation of the immune response and the occurrence of widespread inflammation [59]. Therefore, it is important to effectively mitigate the side effects associated with inflammation, and certain natural products and their active ingredients can achieve this through their anti-inflammatory effects [60]. After exposure to ionizing radiation, the body produces various pro-inflammatory cytokines and chemokines, such as interleukin-1 (IL-1), interleukin-6 (IL-6), tumor necrosis factor-alpha (TNF-α), and transforming growth factor beta (TGF-β) [61]. TGF-β is particularly important in radiation-induced damage, as it mediates tissue fibrosis, such as in the lungs and skin (Figure 6) [62]. Additionally, TGF-β1 activation is induced indirectly by ionizing radiation through the damage to endothelial cells, disrupting the balance of reactive oxygen and nitrogen species [63]. Plant compounds have been studied for their effects on different signaling pathways, such as TGF-β. Natural radioprotectors can help reduce inflammation and tissue damage through a variety of mechanisms [64]. Compounds like resveratrol and crocetin have demonstrated radioprotective effects by attenuating radiation-induced dysfunction in salivary glands and intestinal epithelial cells, respectively [65,66]

## 3. Radiomodulation In Vitro Models

With respect to radioprotective potential, numerous in vitro studies have been conducted on various cell lines. These investigations aim to elucidate the efficacy and mechanisms underlying the radioprotective properties of potential agents. These studies involve exposing different cell types to IR in controlled laboratory settings to assess the ability of specific substances to mitigate the harmful effects of radiation. The outcomes of these in vitro studies contribute valuable insights to our understanding of the radioprotective potential of various compounds. In addition to studying radioprotective potential, in vitro investigations also explore the phenomenon of radiosensitization, particularly concerning its differential effects on healthy and cancerous cell lines. Radiosensitization involves enhancing the sensitivity of cells to IR and potentially increasing the efficacy of radiation therapy in cancer treatment. By observing changes in, e.g., cell survival or DNA damage, the impact of studied compounds or extracts on the radiosensitivity of the cells can be studied (Table 1).

## 4. Radioprotection in Animal Models

Radioprotection studies on animals such as mice, rats, dogs, guinea pigs, minipigs, and rabbits are essential for understanding the impact of radiation on organisms and developing effective health protection strategies. These studies should adhere to the principles of the 3Rs (Replacement, Reduction, and Refinement) and comply with local regulations. To date, numerous animal studies have been conducted. The studies are summarised in Table 2.

### 4.1. Studies Performed on Mice and Rats

The most commonly used animal model for in vivo studies of radioprotective agents is in mice and rats. These animals are small mammals, adaptive to new surroundings, easy to breed, pleasant-natured, procreate fast, and have a short lifespan. They might be used to prove the radioprotective properties of substances, extracts, and oils. In the M.E. assayed study, rats were administered with *Nigella sativa* oil (1 mL/kg b.w.) orally for 5 days a week for four weeks; after the last intubation, animals were gamma irradiated (4 Gy) [82]. In the only radiation-exposed group, rats had leukopenia and exhibited a decrease in total protein concentration in plasma, an increase in malondialdehyde as a lipid peroxidation marker, and a rise in GSHpx activity. All parameters were significantly closer to a normal range in rats treated with black seed oil. P. Uma Devi studied orientin (Ot), vicenin (Vc)- flavonoids from *Ocimum sanctum* leaves, MPG (2-mercaptopropionyl glycine), and WR-2721 as radioprotectants [83]. The potential of the substances was evaluated based on chromosome aberration in mice’s bone marrow cells. Examined animals were divided based on the injected intraperitoneally substances: 50 µg/kg b.w. of Ot or Vc; 20 mg/kg b.w. of MPG; and 150 mg/kg b.w. of WR-2721. Then, after 30 min, mice were exposed to 2.0 Gy gamma radiation. The most effective radioprotectant was WR-2721, and the least effective was MPG. Flavonoids of *Ocimum sanctum* yielded comparable results. In another study, mice were used to evaluate the protective effects against radiation of *T. cordifolia* root alcoholic extract [84]. Increased animal survival was obtained after 90 min of preliminary irradiation with the extract intraperitoneally administrated. According to antioxidation, anti-inflammation, and metal chelation, the extract inhibits lipid peroxidation, radiation-induced micronuclei formation, genotoxicity, and hemopoietic cell injury. More instances of the use of mice and rats are contained in Table 1.

### 4.2. Studies Performed on Dogs

Studies are also performed on dogs. Bradley performed irradiation using neutrons and photons with a mean energy of 15 MeV on beagle dogs [85]. The irradiated fields were the spinal cord, lung, and brain. Nonirradiated and photon-irradiated dogs did not develop any neoplasms. In the group of 46 neutron beam-irradiated dogs, 7 of them developed neoplasms (15%) within the irradiated parts. Also, in 1981, Zook and Bradley partial-body irradiated 39 beagle dogs with photons and neutrons with a mean energy of 15 MeV [86]. After proper thorax irradiation, all dogs developed degenerative and occlusive changes in vessels according to atrial lesions. Beagles died of hepatic failures, neoplasms, and myocardial infarction. The pathologic score of cardiac injury was higher for neutrons than for protons. In another study, beagles were used to determine the radioprotective effects of polysaccharides extracted from *Sipunculus nudus*. Cui I. dogs were orally administered polysaccharides (50, 100, and 200 mg/kg body weight) for seven days and radiated with 2 Gy γ-radiation, and then, for a further 27 days, they maintained polysaccharides administration. After radiation, untreated beagle dogs developed hematopoietic bone marrow damage. In contrast, other dogs stood out with an improved blood picture, hematopoietic activity, a decrease in nitric oxide concentration, and a rise in SOD activity.

### 4.3. Studies Performed on the Guinea Pig Model

Patients after radiotherapy might have sensorineural hearing loss. Some studies have attempted to prevent this in a guinea pig model. The guinea pigs were treated with an intra-peritoneal or intra-tympanic dose of aminothiol PrC-210 (3-(methyl-amino)-2-((methylamino)methyl)propane-1-thiol) a week before inner ear irradiation (3000cGy) [87]. The substance was not cytotoxic and did not impact morphology or the number of cochlear hair cells. One week after irradiation, auditory brainstem response thresholds of the test guinea pig group were better than in the not-injected, irradiated group. The spiral ganglion was efficiently less degenerated in Aminothiol PrC-210-treated animals. Combined in vitro and in vivo study of the potential radioprotective properties of metformin was performed by M. A. Mujica-Mota. In an in vitro study, The House Ear Institute–Organ of Corti 1 (HEI-OC1) cells were administered with 0–5 mM metformin 24 h before 15 Gy irradiation [88]. The guinea pigs were supplemented with metformin dissolved in water to obtain the 100 mg/kg/d dose three days before irradiation and until the end of the experiment [88]. The radiation of 3.5 Gy per day (2.6 min) lasted four weeks from Monday to Friday. Metformin was neither ototoxic nor radioprotective in vitro or in vivo. Orciprenaline and carbachol are radioprotectants at the medicinal treatment dose administered before irradiation of the minipigs [89]. In the study, 36 Gy X-irradiation was administered for six days at 6 Gy for three weeks. Acinar cells were more numerous, and there were significantly fewer pathological changes in the cells.

### 4.4. Studies Performed on Rabbits

Single gamma radiation (4 Gy) on rabbits influences the rabbits’ life duration [90]. Interestingly, irradiated inbred rabbits live shorter lives than outbred ones. Rabbits were fed a standard diet enriched with 100 and 200 ppm of *Yuca schidigera* extract; four weeks later, they were injected with 0.11–0.15 GBq Technetium-99 mIt (emits a single gamma photon) in 1 ml physiological serum [91]. Blood samples were taken before and 10 and 60 min after injection. The rabbits with diet enriched with YSE presented lower malondialdehyde concentrations before and after radiation compared to control rabbits. The concentration of nitric oxide (NO) was only significantly lowered for the rabbits supplemented with a higher concentration of YSE before injection, but after the procedure, there was no significant decrease in the upraised level of plasma NO. The reduction in plasma cholesterol concentration after radiation exposure was faster and more intense in the supplemented group than in the control group of rabbits. There was no significant difference between the levels of and total antioxidant activity in treated and untreated groups. Supplementation of *Yuca schidigera* extract only partially presented antioxidant effects on rabbits exposed to gamma radiation. In the Georgieva et al. [92] study, rabbits were injected with intramuscular 0.24 g/kg *Haberlea rhodopensis* extract 2 h before or 30 min after 2.0 Gy of gamma radiation. The increase after radiation plasma concentration of malondialdehyde as a lipid peroxidation marker was reduced in groups injected with *Haberlea rhodopensis* extract before or after radiation. However, the alkaline comet assay results performed on peripheral lymphocytes showed that only the group previously treated with the extract lowered the irradiation-induced increase in DNA damage frequency. The studies showed that the extract of *Haberlea rhodopensis*, directly and indirectly, has radioprotective properties. Another study, according to Penchev et al. [93], confirms that HRE is a radioprotectant. After a preliminary injection of 0.24 g/kg b.w. of *Haberlea rhodopensis* extract, the tubular diameter and area of the seminiferous tubules escalated compared to only a 2 Gy gamma radiation-exposed group of rabbits. Moreover, the pathological lesions in the tubular structure were less malign in the *Haberlea rhodopensis* extract-treated group of rabbits.

*Macaca mulatta* were gamma irradiated with 60 Co at a lethal dose of 6.8 Gy for 30 min (22.8 cGy/min) [94]. Additionally, 5 min before radiation exposure, juvenile rhesus macaques were administered intramuscularly with 40–120 mg/kg of indralin. Indralin at a dose of 120 mg/kg saved five out of six monkeys from death. It reduced lesions, delayed and reduced the severity of the hemorrhagic syndrome of the disease, and lenified leukopenia and anemia. Indralin is a strong radioprotectant.

**Table 2:** In vivo studies of natural compounds and plant extracts for radioprotection.

Substance/Plant	Application	Radioprotective Dose for Mouse (M) or Rat (R)	Time of Application	Proposed Mechanism
*Aegle marmelos*	Intraperitoneal [95]	15 mg/kg M body weight (optimum)	For 5 days before irradiation	Free-radical scavenging, inhibition of lipid peroxidation, elevation of GSH, and increased activity of antioxidative enzymes
*Ageratum conyzoides*	Intraperitoneal [96]	75 mg/kg M body weight (optimum)	Before irradiation	Scavenging of ROS, increased antioxidant status, and stimulation of the immune system
*Borago officinalis*	Oral [97]	50 mg/kg R body weight	For one week before irradiation and 2 weeks after irradiation	Antioxidant activity, inhibition of MDA, and prevention against GSH depletion
			3 h after irradiation and daily for 2 weeks after irradiation	
Eugenol	Oral [98]	150 mg/kg body weight (optimal)	3 h after irradiation and daily for 2 weeks	Protection against oxidative stress, induction of detoxifying enzymes, scavenging of free radicals, inhibition of lipid peroxidation
Genistein	Subcutaneous [99]	200 mg/kg body weight (optimal)	24 h before irradiation	Estrogenic activity, antioxidant properties, immunostimulatory activity and its role in signal transduction pathways where it is an inhibitor of topoisomerase, protein kinase and caspases involved in apoptotic pathways, cytokine release
	Oral [100]	160 mg/kg M body weight	For seven consecutive days before irradiation	
*Glycyrrhiza glabra*	Intraperitoneal [101]	4 mg/kg M body weight	Before irradiation	Scavenging of free radicals
*Hippophae rhamnoides*	Intraperitoneal [102]	30 mg/kg M body weight	30 min before irradiation	Free-radical scavenging, acceleration of stem cell proliferation, and immunostimulation
*Curcumin*	Oral [103]	20 mg/kg M body weight (optimal)	2 h before irradiation and 24/30/48 h after irradiation	Scavenging of free radicals and the elevation of cellular antioxidants, upregulation of CAT, glutathione transferase (GST), GSHpx, SOD, and their mRNAs, reduction in lipid peroxidation, elevation in GSH and increase in sulphydryl groups, inhibition of activation of Protein Kinase C (PKC), Mitogen-Activated Protein Kinase (MAPK), and NO [k’]
*Folic acid*	Intraperitoneal [104]	1.6 mg/kg M body weight	For 10 days after irradiation	Scavenging of free radicals, mainly by peroxynitrite scavenging and lipid peroxidation inhibition
*luteolin*	Oral [105]	10 µmol/kg M body weight	2 h before irradiation	Scavenging potency towards free radicals
Orgotein	Subcutaneous [106]	400 mg/kg M body weight (optimal)	1 to 2 h before irradiation	Anti-inflammatory action of the drug
*Panax ginseng*	Intraperitoneal [107]	50 mg/kg M body weight	36 and 12 h before irradiation	Antioxidant and free-radical-scavenging activities of the ginsenosides
	Intraperitoneal [108]	10 mg/kg M body weight	For 4 days before irradiation	Inhibition of initiation of free-radical processes by antiradical actions, e.g., inhibition of lipid peroxidation
Resveratrol	Intraperitoneal [109]	50 and 100 mg/kg M body weight	2 h before irradiation	Antioxidant mechanisms
	Oral [110]	20 mg/kg M body weight (optimal)	For 7 days before irradiation and 30 days after irradiation	Antioxidant properties: scavenging free radicals, regulation of the redox of a cell by differentially affecting the expression of various oxidases and antioxidant enzymes
Rutin	Oral [111]	10 mg/kg M body weight (optimal)	For 5 days before irradiation	Scavenging of free radicals resulting in decreased oxidative stress in animals, normalization of intracellular antioxidant levels, anti-lipid peroxidative effect
Quercetin	Oral [111]	20 mg/kg M body weight (optimal)	For 5 days before irradiation	Scavenging of free radicals resulting in decreased oxidative stress in animals, normalization of intracellular antioxidant levels, anti-lipid peroxidative effect

## 5. Forms of Administration of Radioprotectants

Substances of radioprotective importance can be applied both topically and orally. Each method has advantages and disadvantages, and scientific studies have considered both.

Several factors must be considered when developing a topical preparation with radioprotective effects. The advantages of such a formulation include targeted delivery to the affected area, potentially reducing systemic side effects, and providing a more localized protective effect against radiation-induced damage [112,113]. Additionally, the use of topical formulations can offer better patient compliance and ease of application, particularly in scenarios where oral administration may be challenging, such as in cases of oral mucositis resulting from radiation therapy [114,115]. Furthermore, the potential for controlled drug delivery and the ability to maintain stable drug formulations in the solid state can be advantageous for topical oral preparations, especially in scenarios where storage at ambient temperature and longer shelf-life are crucial [116,117].

However, developing a topical preparation with radioprotective effects also presents challenges. Ensuring the sterility of the formulation is critical, particularly for burn wounds, to prevent infections and promote effective wound healing [118,119]. Additionally, the formulation must be designed to penetrate the skin barrier effectively to reach the target tissues and provide the desired radioprotective effects. Furthermore, the potential for microbial contamination in burn wounds necessitates using antimicrobial agents in the formulation to prevent infections and promote healing. Moreover, the formulation should be designed to minimize disruption of the body’s natural healing mechanisms and maintain a moist, aseptic environment to support optimal wound healing. The use of topical preparations containing colored plant compounds can also disrupt the image of the wound.

Also, oral delivery of radioprotective agents presents advantages and disadvantages in protecting against radiation-induced damage. One significant advantage is the potential for systemic protection. Oral administration of radioprotective agents allows for the distribution of protection throughout the entire body, safeguarding multiple organs and tissues simultaneously [120,121]. This systemic approach is crucial in mitigating the broad impact of radiation exposure. Another notable benefit is enhanced patient compliance. Oral administration offers a familiar and convenient drug delivery route, potentially improving patient adherence to radioprotective treatment regimens [121]. The accessibility of oral delivery may contribute to better patient cooperation, a crucial factor in the success of radiation protection strategies.

Moreover, the versatility of oral formulations allows for targeted delivery to specific tissues or organs. This targeted approach enables localized protection against radiation-induced injury, offering a more tailored and efficient protective mechanism [120,122]. Eliminating procedures, such as using ointments and gels, is another advantage. Additionally, oral formulations can be designed for controlled release, providing sustained and prolonged radioprotective effects. This controlled-release feature adds to the efficacy of oral delivery, ensuring a more consistent and prolonged protection against radiation exposure.

However, the oral delivery of radioprotective agents has its challenges. One notable drawback is the variability in drug absorption, leading to inconsistent bioavailability and potentially affecting the overall efficacy of radioprotective agents [120]. Gastrointestinal side effects, such as nausea, vomiting, and diarrhea, are associated with oral administration and can impact patient tolerance and compliance. First-pass metabolism in the liver poses another challenge. Orally administered drugs undergo this metabolic process, which can result in reduced bioavailability and efficacy of radioprotective agents [123]. Overcoming the hurdles of first-pass metabolism is essential for optimizing the protective effects of orally delivered radioprotective agents. Furthermore, oral formulations may exhibit a delayed onset of action compared to parenteral routes, potentially limiting their effectiveness in acute radiation exposure scenarios. In situations requiring immediate response, this delay could be a critical factor to consider.

In summary, while oral delivery of radioprotective agents offers systemic protection, patient compliance, and potential for targeted delivery, it is not without challenges. The variability in absorption, gastrointestinal side effects, first-pass metabolism, delayed onset of action, and sterility requirements may impact the overall efficacy of this delivery method. Careful consideration of these factors is essential in determining the suitability of oral administration for radioprotective strategies.

## 6. Compounds of Natural Origin Showing Radioprotective Activity

### 6.1. Terpenes

A large group of hydrocarbons is terpenes. These substances are built up from isoprene units. According to the number of units, we can differentiate monoterpenes (C10H16), sesquiterpenes (C15H24), diterpenes (C20H32), and so on. Similarly named, terpenoids stand for substances that have been terpenes derivates, such as naturally oxidated or chemically modified terpenes.

**Figure 7:**
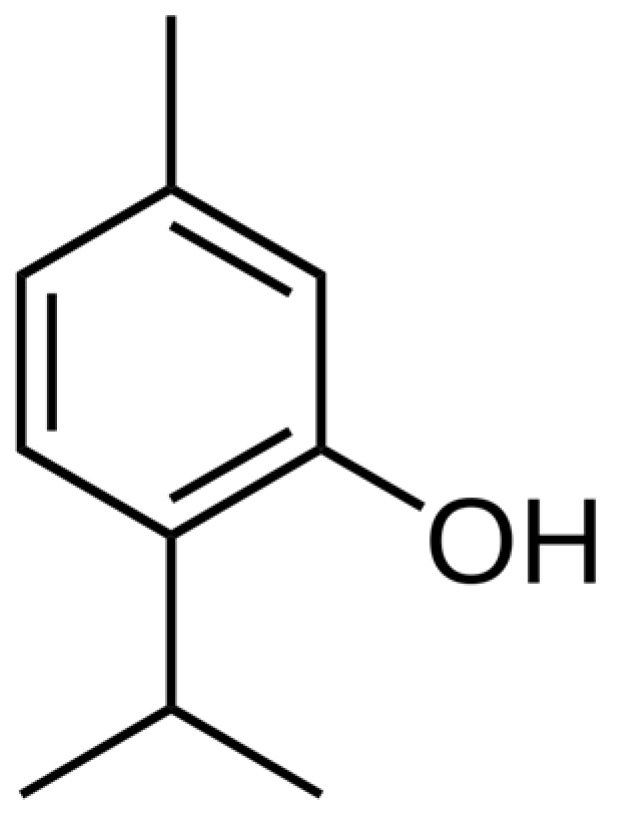
The structure of thymol.

Carvacrol and thymol (Figure 7), known for their antioxidative, anti-inflammatory, and radioprotective properties, demonstrated nephroprotective potential in rats subjected to gamma irradiation. The findings suggest these compounds may alleviate acute RN by mitigating oxidative stress, inflammation, and apoptosis. Additionally, the preservation of the Calcitonin Gene-Related Peptide-Tumor Necrosis Factor Alpha (CGRP-TNF-α) loop and the in silico molecular docking simulations support the notion that carvacrol and thymol could enhance the therapeutic benefits of gamma irradiation [124]. In rats, thymol (present, e.g., in *Thymus* sp.) showed significant protection in acute and chronic post-radiation damage in salivary glands [125]. According to Sueishi et al. [126], menthol (*Mentha piperita*) is the strongest radioprotector of the three monoterpenes linalol, thymol, and menthol. A study conducted by Mahran et al. [127] demonstrates the radioprotective effects of carvacrol and thymol, particularly in rescuing ovarian reserve through counteracting oxidative stress and dysregulated cross-talk between (Insulin-like Growth Factor 1) IGF-1 and TNF-α.

### 6.2. Carotenoids

**Figure 8:**
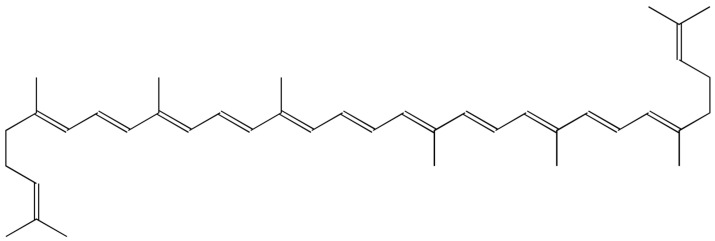
The structure of lycopene.

Carotenoids are pigments produced by plants. They are the most commonly known to be responsible for the color of tomatoes, carrots, pumpkins, corn, and bananas. We differentiate two classes: oxygen-containing xanthophylls and carotenes. In the first group, we can include lutein and zeaxanthin. We can assign α-carotene, β-carotene, and lycopene to the no-oxygen class. According to Vasudeva et al. [128], lutein showed protection over cytogenetic damages induced by radiation in Swiss albino mice; it also has antioxidant and anti-inflammatory properties. Lycopene (Figure 8) is another carotenoid that manifests radioprotective properties. Pretreatment of mice with lycopene before irradiation reduced radiation sickness and prolonged the lifespan of the animals. Lycopene increased the level of hepatic GSH, GSHpx, and SOD in irradiated rats and also played a protective role for cultured human lymphocytes [129,130]. In the group of carotenoids, there is also a radiosensitizer, crocin, which is a compound of saffron. In the study on head and neck cancer cell line (HN-5), the cells were administered crocin in different concentrations (12.5–1000 µg/mL) and were then irradiated. The substance reduced cell viability, induced apoptosis, and sharpened radiation’s effect [131]. The survival rate of cells treated with crocin is comparable to that of cells treated with amifostine—the only radioprotector clinically approved by the US Food and Drug Administration (FDA) after irradiation with a dose of 6 Gy. Crocin resulted in significant prevention of radiation-induced damage in peripheral blood cells.

### 6.3. Flavonoids

**Figure 9:**
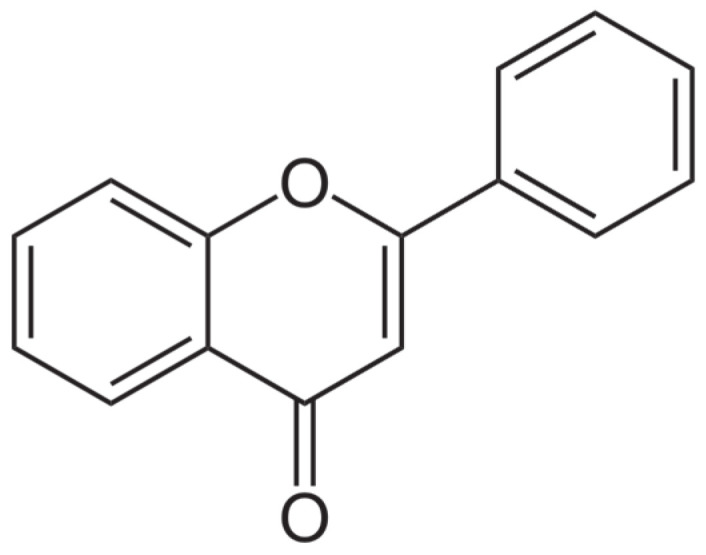
The general structure of the 15-carbon skeleton of flavonoids.

Flavonoids have a structure of a 15-carbon skeleton that consists of two benzene rings (Figure 9). They might occur in a free form as hydrophobic aglycone or as a combination of aglycone with a sugar–glycoside (C-glycoside or O-glycoside), which is soluble in water. They are present in many fruits and vegetables, green and black teas, red wine, beans, and grains. They have an influence on the human body in various directions. Flavonoids are anti-inflammatory, antibacterial, antimutagenic, antioxidant, and helpful in treating cardiovascular diseases [26,132]. They inhibit cyclo-oxygenase, lipoxygenase, acetylcholinesterase, and butyrylcholinesterase [133,134,135]. Depending on the structure, flavonoids consist of flavanones, flavones, flavonols, isoflavonoids, and chalcone.

#### 6.3.1. Hesperidin

**Figure 10:**
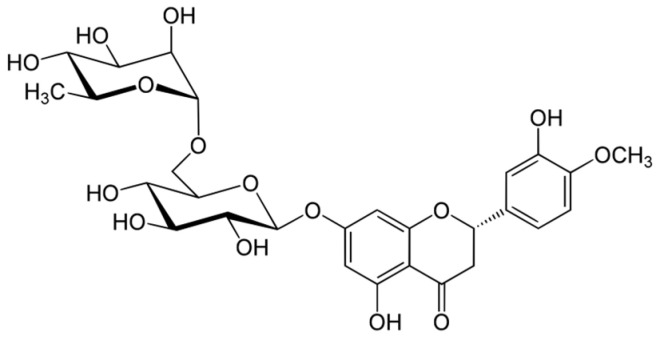
The structure of hesperidin.

A flavanone, hesperidin (Figure 10), is found in citrus fruits. The administration of hesperidin decreased inflammation, fibrosis, and myocyte necrosis in rats [13,136]. Unfortunately, it did not reduce myocardial degeneration or vascular leakage [13].

The article by Musa et al. [137] provides a comprehensive review of the potential radioprotective effects of hesperidin. The systematic review explores hesperidin’s antioxidant, anti-inflammatory, and antiapoptotic abilities as a potential radioprotective agent against IR-induced damage. In one of the studies on mice, liver hesperidin was administered intragastrically before irradiation, which increased the level of antioxidant enzymes and reduced DNA damage [138]. The wounds on irradiated mice contract later, slower, and healing takes longer. The synthesis of fundamental wound healing components, DNA, collagen, hexosamine, and NO were declined. Pretreatment of mice with (100 mg/kg body mass) hesperidin before γ-radiation resulted in faster recovery of mice wounds; it contracted more, and it shortened the time of the process. The synthesis of essential wound healing factors determined in the study was increased in these mice. Moreover, the density of blood vessels and fibroblasts in the wounds was greater in hesperidin-treated mice [139]. The volunteers took 250 mg of hesperidin once; then, the blood was withdrawn at different time points and irradiated. Every blood sample had an increased level of micronuclei; however, hesperidin intake before irradiation reduced it. The most efficient change was reported for the blood sample withdrawn one hour after hesperidin consumption. The results underline the possibility of lymphocyte protection against genotoxicity with hesperidin [140]. In the study conducted by Haddadi et al. [136], the initiation of angiogenesis was observed through the induction of the VEGF gene. This process stimulated epithelialization, promoted collagen deposition, and enhanced cellular proliferation. These effects collectively contributed to the facilitation of wound healing and provided protective mechanisms against radiation-induced damage to the skin.

#### 6.3.2. Apigenin

**Figure 11:**
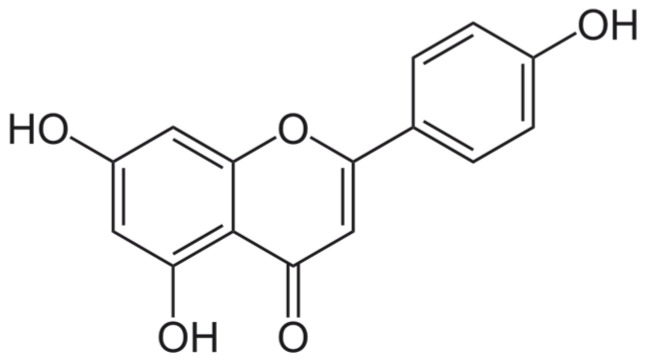
The structure of apigenin.

In the subgroup of flavones, apigenin (Figure 11) is a ROS scavenger; it reduces clastogenic post-radiation effects and decreases hematological damage [44,45]. The human peripheral blood lymphocytes were treated with apigenin an hour before irradiation. It reduced the ROS level and decreased the apoptosis frequency in cells. Moreover, it is opposed to decreased mitochondrial membrane potential in human peripheral blood lymphocytes. It influences antioxidative enzymes—apigenin increases the level of GSH, CAT, and SOD. It protects from lipid peroxidation and micronuclei formation in irradiated lymphocytes.

Furthermore, it enhanced the expression of Bcl-2 and lessened the expression of p53, p21, Bax, and NF-kB. As a potent antioxidant, apigenin protects human peripheral blood lymphocytes from radiation-induced injuries [56]. In a study on radiation-induced intestinal injury (RIII), apigenin, a natural flavone, has significantly improved survival in C57 mice after lethal irradiation [141]. Apigenin pretreatment expedited the restoration of the crypt–villus structure, enhancing crypt regeneration, epithelial cell differentiation, and increased villus length. Mechanistically, apigenin demonstrated neuroprotective potential by upregulating Nrf2 and HO-1, reducing oxidative stress, and promoting intestinal crypt cell proliferation while inhibiting apoptosis.

#### 6.3.3. Tangeretin

**Figure 12:**
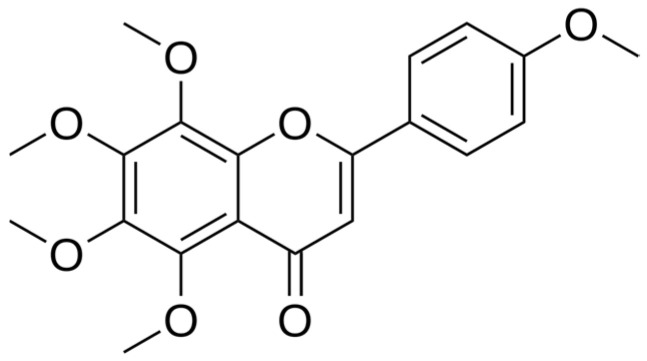
The structure of tangeretin.

Tangeretin (Figure 12) is an antioxidant that induces apoptosis in human leukemia cells. It upregulates a suppressor of tumor microRNA—miR-410. Moreover, repeated expression of miR-410 results in counteraction of radiation-induced epithelial–mesenchymal transition. Tangeretin is a radiosensitizer of gastric cancer cells and an antimetastasis agent [142]. Luteolin is a potent antioxidant; it upregulates Bax and decreases the expression of Bcl-2. It induces apoptosis in HT-29 colon cancer cells by promoting antioxidant properties and activating MAPK signaling. It increases the level of GSH and antioxidative enzymes like synthetase GSH and CAT [143]. In the study, the mice were intragastrically administered luteolin (5 mmol/kg) 6 h before γ-radiation [144]. Next, the micronucleated reticulocytes in peripheral blood were measured. Luteolin turned out to be an antioxidative and anticlastogenic agent. Surprisingly, luteolin generates ROS after exposure to X-ray and UV radiation like O2 [145].

#### 6.3.4. Quercetin

**Figure 13:**
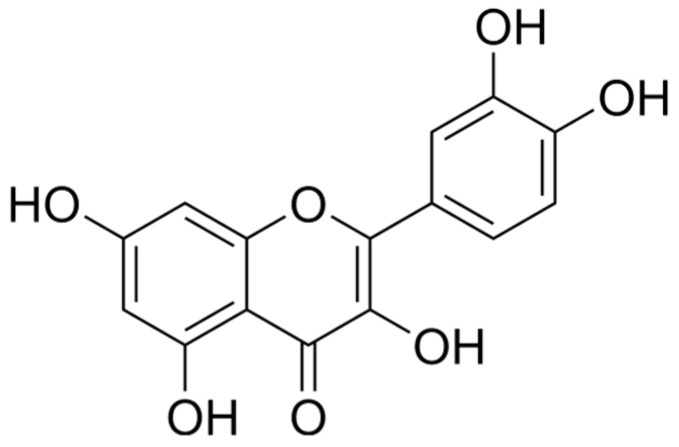
The structure of quercetin.

Flavonols like quercetin (Figure 13) and glucosides, such as rutin, are radioprotective factors. Pretreatment of mice with these substances before gamma radiation resulted in the elevation of the level of antioxidative enzymes like GSH, CAT, and SOD, and lipid peroxidation was reduced [146]. Moreover, quercetin protects white blood cells and DNA from post-radiation damage [147]. In the in vitro study, pretreatment of lymphocytes with rutin before gamma radiation reduced the number of micronuclei protecting white blood cells. There was also a decline in DNA damage according to the determination of percent tail DNA and olive tail moment [146]. Rutin’s derivate, troxerutin, administered prior to gamma irradiation increased the activity of antioxidative enzymes of the liver and reduced the damage that radiation caused to the organ [148]. Troxerutin reduces radiation-induced lipid peroxidation in the liver and spleen in normal cells; it also protects from DNA damage according to the determination of the percentage of DNA in white blood cells and bone marrow [149]. The 30-day survival after gamma irradiation was determined in the study conducted in mice. Troxerutin turned out to be a radioprotector depending on the dose; it significantly elevated the survival of the animals [150].

### 6.4. Isoflavonoids

**Figure 14:**
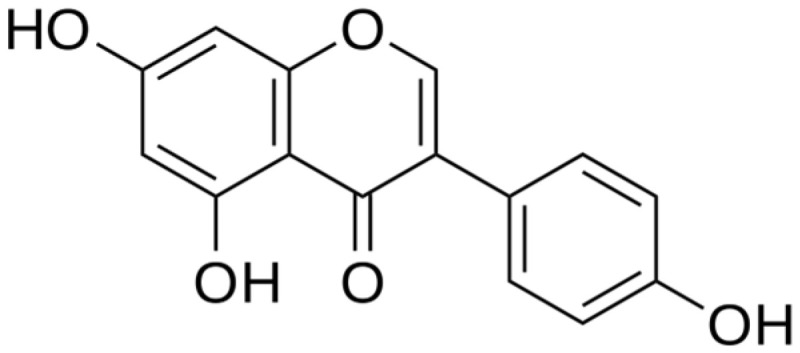
The structure of genistein.

Genistein, an isoflavonoid (Figure 14), shows dose-dependent effects on the human liver cell line. In concentrations of 1.5 µM, as a radioprotectant, it inhibited apoptosis and reduced DNA damage, and in concentrations of 20 µM and 40 μM, it played the role of radiosensitizer [151]. Moreover, genistein administered 24 h before gamma irradiation in mice elevated the survival of the animals [99]. The isoflavonoid reduced the increase in breathing rate in rats post radiation during early pneumonitis. Moreover, the animals survived longer than just the irradiated group. It also declined TNF-α, IL-1β, and TGF-β levels and protected rats’ DNA. However, treatment with genistein did not prevent animals’ death; it only delayed it. Genistein at 200 mg/kg administered to mice one day prior to irradiation resulted in a reduction in tubular atrophy compared to the only radiated group. Moreover, the post-radiation elevated malondialdehyde level was lowered due to genistein [152].

### 6.5. Anthocyanins

**Figure 15:**
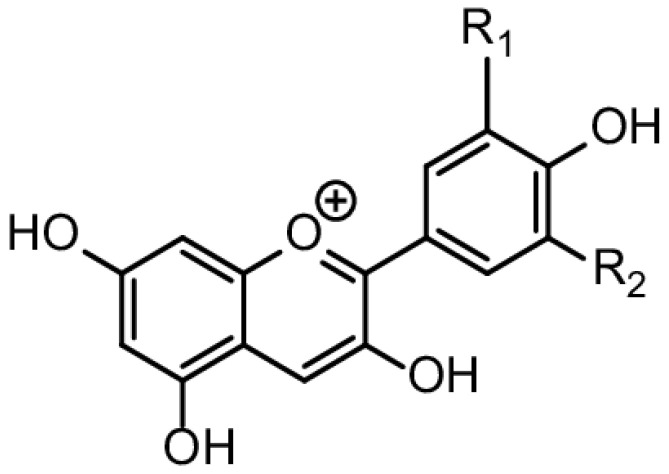
The structure of anthocyanins.

Anthocyanins, a type of phytochemical found in various fruits (Figure 15), have been studied for their potential radioprotective effects. Fan et al. [153] demonstrated that anthocyanin-rich extracts from lingonberries effectively mitigated radiation-induced damage in mice without causing acute toxicity. Similarly, anthocyanins extracted from *Vaccinium Vitis-idea* L fruits showed radioprotective properties in a preclinical in vivo study [154]. Moreover, the anthocyanins present in date syrup were attributed to its radioprotective effect on DNA, along with other constituents like proanthocyanidins, β-carotene, and selenium [155].

Studies have also explored the radioprotective effects of anthocyanins against different types of radiation. While Fan et al. [153] focused on IR, Kim et al. [69] mentioned the radioprotective effects of delphinidin, a type of anthocyanin, against proton beam exposure.

Overall, anthocyanins have shown promise in providing radioprotective benefits, potentially due to their antioxidant properties. These compounds have been associated with mitigating radiation-induced damage, protecting against DNA double-strand breaks, and enhancing cell survival post radiation exposure. The diverse sources of anthocyanins, ranging from lingonberries to *Vaccinium Vitis-idaea* L fruits, highlight the potential of these phytochemicals in offering radioprotective effects. The antioxidant activity of anthocyanins involves processes such as hydrogen atom transfer (HAT) or single-electron transfer (SET), enabling them to scavenge radiation-induced ROS [69]. These mechanisms help mitigate oxidative stress and DNA damage caused by radiation exposure, contributing to the radioprotective characteristics of anthocyanins.

Moreover, the radioprotective effects of anthocyanins may involve modulating inflammatory responses and cellular toxicity induced by radiation. Anthocyanins act as radioprotective agents by targeting the oxidative stress response and inflammation pathways, enhancing cellular resilience against radiation-induced damage [156,157]. Encapsulating anthocyanins in nanocarriers enhances their radioprotective capabilities by prolonging their retention time and ensuring sustained protection against radiation-induced oxidative stress [158].

### 6.6. Tannins

**Figure 16:**
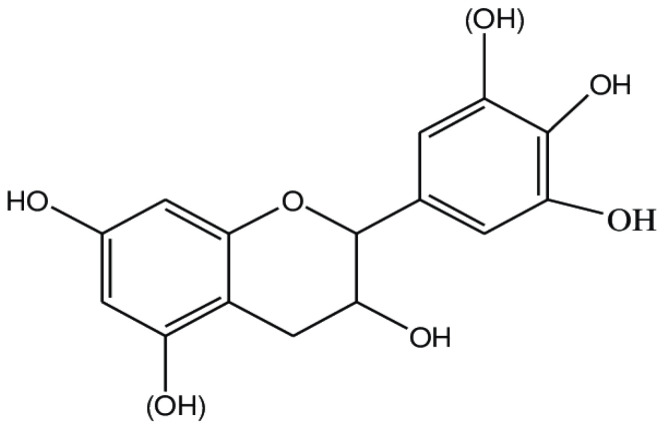
Structure of flavonoid unit of condensed tannins.

Tannins are polyphenolic (Figure 16), water-soluble compounds that precipitate proteins and amino acids. They have antimicrobial, anti-inflammatory, and antimutagenic properties and they might be helpful in a topical application on the skin according to their astringency [159,160]. Oral administration of 100 mg/kg body weight of gallic acid to mice before gamma irradiation increased the level of GSH, decreased DNA damage, and inhibited lipid peroxidation [161]. Persimmon tannin from *Diospyros kaki* L.f. was studied in the radioprotective direction. Pretreatment of human embryonic kidney 293T cells with persimmon tannin before gamma-radiation increased cell viability, reduced apoptosis, and reduced ROS levels [162]. Chinese hamster lung fibroblasts were gamma radiated, but before that, they were pretreated with geraniin, another tannin, isolated from *Nymphaea tetragona* [163]. The tannin reduced the level of ROS, increased the activity of antioxidative enzymes such as SOD and CAT, and reduced cell apoptosis. Substances from this group may be radioprotectants and radiosensitizers like ellagic acid. Ellagic acid increases oxidative stress in cancer cells. After administration of 10 µM of the substance 12 h before the radiation of the human hepatocellular carcinoma cell line—HepG2, the generation of ROS was increased, it upregulated p53, apoptosis of the cells was increased, and the survival markers were worse, like phosphorylated Akt, phosphorylated NF-kB, and phosphorylated STAT3, compared to only radiated cells. Combining radiotherapy with ellagic acid administration might result in using lower doses of radiation [164].

The quinones are cyclic, unsaturated organic compounds that are known for aperient, antimicrobial, and antiparasitic properties; they also have antioxidant and antitumor activity, and they might prevent cardiovascular diseases and osteoporosis [165,166,167]. Pyrroloquinoline quinone, which presents naturally in soil and kiwifruit, takes part in a variety of physiological processes such as cellular energy metabolism, antioxidation, redox reactions, and mitochondrial biogenesis in muscles [168]. Pyrroloquinoline quinone showed radioprotective activity on X-ray irradiated mice on their parotid glands due to reduction in DNA damage and ROS scavenging and inhibition of apoptosis [169]. In another study, administering pyrroloquinoline quinone to mice accelerated the recuperation of leukocytes, reticulocytes, and bone marrow cells after radiation [170]. The quinone increased the survival of gamma-radiated mice and expedited hematopoietic recovery. In vitro gamma radiation enhanced lipid peroxidation and increased nitric oxide and hydroxyl radicals. However, hydroquinone inhibited the process and scavenged the harmful radicals [171].

### 6.7. Alcaloids

**Figure 17:**
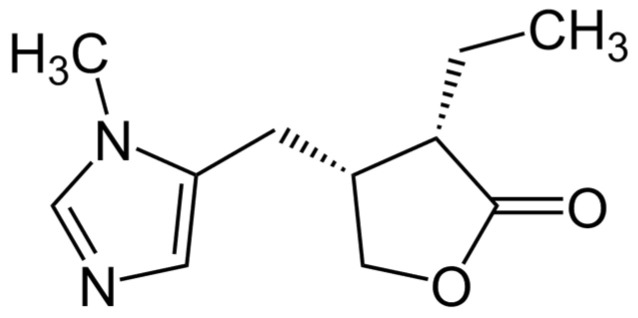
The structure of pilocarpine.

Plant alkaloids, a diverse group of natural compounds, can be classified into various classes based on their chemical structures (Figure 17). These include indole alkaloids, isoquinoline alkaloids, tropane alkaloids, quinoline alkaloids, pyrrolidine alkaloids, piperidine alkaloids, and purine alkaloids. Each class exhibits unique properties, contributing to the pharmacological diversity of these plant-derived compounds [172]. Pretreatment of mice with sanguinarine (present, e.g., in *Sanguinaria* sp., *Argemone* sp., and *Chelidonium majus*) prolonged the survival of animals after radiation. Moreover, it decreased intestinal and lung damage [173]. Intake of the imidazole derivate pilocarpine (*Piolocarpus* sp.) helped patients to increase salivary flow and decrease xerostomia [174]. The most commonly known alkaloid, caffeine, is present in coffee, tea, and cacao.

### 6.8. Coumarins

**Figure 18:**
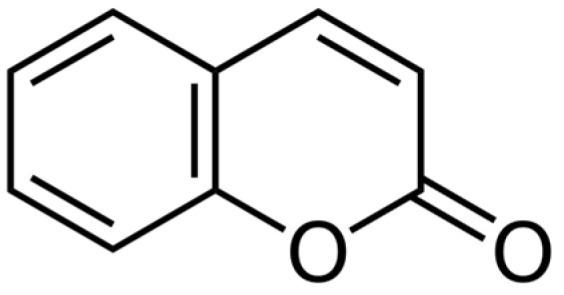
The basic structure of coumarins.

Coumarins classified as benzopyrone derivates (Figure 18) are poorly soluble in water but soluble in alcohol and lipids. In vitro, pretreatment with umbelliferone in isolated human blood lymphocytes inhibited ROS, increased the activity of antioxidant enzymes, and reduced lipid peroxidation apoptosis [175]. The coumarin isofraxidin shows radioprotective properties in human leukemia cell lines by decreasing ROS generation; it is opposed to the decrease in mitochondrial membrane potential; and it reduces the expression of Bax in mitochondria [176]. The antiangiogenic synthetic isocoumarin, NM-3, combined with radiotherapy in mice with Lewis lung carcinoma, reduces the tumor volume compared to radiation alone [177].

The radiochemical study of the antioxidant activity of biologically essential compounds from plant materials revealed that unsubstituted coumarin exhibited more significant radioprotective activity [178]. It additionally demonstrated the radioprotective potential of interruptin C, a coumarin-derived compound, in protecting normal breast MCF-10A and HaCaT cells against radiation-induced damage [70]. Furthermore, researchers investigated the inhibitory effect of coumarin on the germination of ryegrass and its potential application as coumarin–carbon dots nanocomposites, suggesting its potential as an herbicidal agent [179].

Moreover, it highlighted the antioxidant activity of coumarin, emphasizing its potential role in combating radiation-induced inflammation [180]. It additionally explored the radioprotective effect of whey hydrolysate peptides against γ-radiation-induced oxidative stress in BALB/c mice, providing insights into the potential radioprotective properties of coumarins [181]. Furthermore, Sharapov et al. [182] investigated the radioprotective role of peroxiredoxin 6, shedding light on its potential mechanisms of action.

## 7. Plant Materials

### 7.1. Glycyrrhiza glabra

*Glycyrrhiza glabra*, family *Fabaceae*, comes from South Europe and Asia. It is an expectorant, antiulcer, slightly laxative, antioxidant plant [183]. The plant might be used to prevent and treat oral mucositis connected with radiation cancer treatment [184]. The study by Mamgain et al. [185] investigates the efficacy of *Glycyrrhiza glabra* on radiation-induced mucositis in head-and-neck cancer patients. *Glycyrrhiza glabra* was observed to be effective and delayed the development of a severe form of mucositis. Glycyrrhizin, derived from licorice root, demonstrates significant protective effects against radiation-induced damage in submandibular glands (SMGs). It reduces oxidative stress, downregulates high mobility group box-1/ toll-like receptor 5 (HMGB1/TLR5) signaling, and preserves mitochondrial integrity, ultimately inhibiting apoptosis. Glycyrrhizin could serve as a promising mitochondria-targeted antioxidant to prevent radiation sialadenitis in head-and-neck cancer patients undergoing radiotherapy [186]. The root extract decreased lipid peroxidation in microsomal membranes of rat liver induced by gamma radiation [187]. Albino rats treated with licorice and then irradiated presented counteraction to radiation-induced decrease in GSH, SOD, and CAT; it also decreased the level of triglyceride and total cholesterol [188]. Glycyrrhizic acid protects human leukocytes from gamma radiation-induced DNA damage, depending on the concentration of the substance. Moreover, in the study on mice administered intraperitoneally with glycyrrhizic acid, the compound counteracted the strand break in the DNA of white blood cells and bone marrow cells [189]. Silver nanoparticles, in conjunction with glycyrrhizic acid, exhibit enhanced capability in safeguarding cellular DNA from harm caused by IR. The heightened protective efficacy of this compound, in contrast to individual silver nanoparticles or glycyrrhizic acid, may be attributed to the combined free-radical scavenging properties inherent in both glycyrrhizic acid and silver nanoparticles within the complex [190].

### 7.2. Aloe barbadensis

*Aloe barbadensis,* family *Asphodelaceae*, can be found in dry regions of various continents: Africa, America, Asia, and Europe. The plant is commonly used in cosmetics. It has healing, moisturizing, and anti-inflammatory properties according to the polysaccharide, auxin, and gibberellin content, and is a laxative due to antra-compounds, and has antiseptic properties due to saponins, salicylic acid, and lupeol [191]. *Aloe vera* leaf ethanolic extract administered in mice decreased lipid peroxidation and increased the concentration of reduced GSH after gamma radiation due to antioxidant and radical-scavenging properties [192]. In patients, a lotion containing *Aloe vera* decreases post-radiation inflammation of the skin [193]. To investigate the potential radioprotective effects of Aloe vera, Dadupanthi et al. [194] focused on the hepatosomatic index of Swiss albino mice. The research concluded that *Aloe vera* extract could indeed offer protection against radiation-induced oxidative stress, suggesting its ability to mitigate the harmful effects of radiation therapy.

Similarly, Nejaim et al. [195] examined the radioprotective properties of *Aloe vera* and zinc/copper compounds on salivary dysfunction in irradiated rats. Their findings supported the effectiveness of *Aloe vera* extract in treating radiation-induced burns, indicating its potential as a radioprotective agent. Furthermore, Liu et al. [196] explored the impact of a persimmon tannin–*Aloe vera* composite on cytotoxic activities and radioprotection against X-ray irradiation in specific cells. The tested composite shows promising radioprotective properties, protecting liver cells (L02 and HepG2) from the harmful effects of IR. PT-A effectively reduces apoptosis and produces ROS, showing better efficacy than the single components, persimmon tannin and *Aloe vera*.

### 7.3. Mentha piperita

*Mentha piperita*, family *Lamiaceae*, is a cross between *Mentha aquatica* and *Mentha spicata*, which nowadays is familiar and widespread. The most frequent use of the leaves is for gastrointestinal tract problems; they have anti-inflammatory, antioxidant, and antimicrobial properties [197,198]. Research by Jagetia and Baliga et al. [199] investigated the impact of *Mentha arvensis* leaf extract on the survival of mice exposed to varying doses of gamma radiation. The study demonstrated a positive effect on the survival rate of mice, suggesting that mint extract could protect against the harmful effects of radiation exposure. In another study, post-radiation damages were alleviated by *M. piperita* extract. The number of red and white blood cells was elevated, the level of GSH was raised, lipid peroxidation was reduced, and the weight of animals was increased compared to only radiated animals [200]. The radioprotective properties of *Mentha piperita* leaf extract were studied in mice testis, and its weight was raised in the group of animals not only radiated but also pretreated with extract; there was a normalization of the level of alkaline phosphatase in tests, the damage in the seminiferous epithelium was smaller in this group [201]. *Mentha piperita* administered before radiation in mice also decreased chromosomal damage in the bone marrow and increased the activity of antioxidant enzymes [202]. Not only is *Mentha piperita* a radioprotector, but *Mentha arvensis* also plays this role. The administration of 10 mg/kg of mint chloroform extract to mice exposed to gamma radiation prolonged the life of the animals. It protected mice from gastrointestinal and bone marrow deaths. The substance is not toxic up to the concentration of 100 mg/kg body mass [199].

The radioprotective mechanism of mint, particularly *Mentha arvensis*, has been explored in a study by Baliga and Rao [203]. The research suggests that the radioprotective effects of mint may be attributed to various factors, including free radical scavenging, antioxidant properties, metal chelation, anti-inflammatory effects, antimutagenic activity, and enhancement of DNA repair processes. These mechanisms collectively contribute to the ability of mint to protect against radiation-induced damage and mitigate the complications associated with radiation therapy. By scavenging free radicals, mint may help reduce oxidative stress and cellular damage caused by radiation exposure, offering a natural and effective approach to enhancing the safety and efficacy of radiation therapy.

### 7.4. Rosemarinus officinalis

*Rosemarinus officinalis* extract administered to mice for five days before gamma irradiation prolonged the lifespan of the animals. The most efficient dose was 1000 mg/kg body weight. The extract reduced lipid peroxidation and increased the level of GSH in the blood and liver after 8 Gy gamma radiation [204]. Rosemary leaf extract was dispensed in the concentration of 1000 mg/kg to Swiss albino mice, which were later exposed to 3 Gy gamma radiation. The extract-treated animals’ number of erythrocytes, white blood cells, hematocrit percentage, and hemoglobin content were elevated compared to the only radiated group. Furthermore, the GSH level was increased, and lipid peroxidation was decreased in the experimental group [204].

### 7.5. Ficus racemosa

The next plant, *Ficus racemosa*, family *Moraceae*, is used in Ayurveda. There were reported anti-inflammatory, antioxidant, hepatoprotective, and cardioprotective properties [205]. In vitro and in vivo extracts of *F. racemosa* decreased radiation-induced DNA damage. This extract inhibited activated post-radiation protein p53, which induces apoptosis [206]. Ethanol extract of *Ficus racemosa* shows antioxidative properties by scavenging superoxide and hydroxyl, 2,2′-Azino-bis(3-ethylbenzothiazoline-6-sulfonic acid) (ABTS•−), 2,2-Diphenyl-1-picrylhydrazyl (DPPH) radicals and inhibiting lipid peroxidation. Moreover, in the study on Chinese hamster lung fibroblast cells (V79), administration of an extract of *Ficus racemose* 1 h before γ-radiation decreased the number of micronucleated binuclear cells [207].

### 7.6. Ginkgo biloba

*Ginkgo biloba* leaf extract was administered to rats at 40 mg/kg for three days prior to 5 Gy gamma radiation and seven days after. The activity of antioxidant enzymes glutathione reductase and GST was raised, and the scavenging of radicals was noticed compared to the only radiated group of animals [208]. *Ginkgo biloba* extract (50 mg/kg daily) dispensed to rats for 15 days prior to irradiation prevented the lungs, liver, and kidneys from radiation-induced damage. It reduced malondialdehyde and myeloperoxidase activity and increased the GSH level [209]. Another study studied the radioprotective effect of fermented and not-fermented *Ginkgo biloba* leaf extract. Both extracts administered for 15 days to rats before gamma irradiation counteracted the decrease in SOD, GSHpx activity, the enlargement of calcium levels in the brain cytosolic fraction, malondialdehyde concentration, and stress hormones levels like epinephrine, norepinephrine, and dopamine. However, fermented *Ginkgo biloba* leaf extract was more efficient in preventing radiation-induced damage [210].

### 7.7. Hippophae rhamnoides

*Hippophae rhamnoides*, family *Elaeagnaceae*, occurs in Europe and Asia. In traditional medicine, the juice is used to heal wounds and treat ulcers, whereas oil extracts are used in skin disorders [211,212]. In gamma-irradiated rats, leaf extract decreased the level of corticosterone in plasma and the level of serotonin in both jejunum and plasma [213]. Pretreatment of mice with *Hippophae rhamnoides* berry extract increased the weight of the testis and the sperm count after gamma radiation. The extract might also prevent post-radiation DNA damage [214]. A fraction of *Hippophae rhamnoides*, rich in flavonoids, has antioxidative properties, scavenges free radicals, and has a significant potential to protect membranes. These properties were ascribed to quercetin, kaempferol, and isorhamnetin. The fraction was not toxic up to 200 mg/kg [215].

### 7.8. Ocimum sanctum

*Ocimum sanctum*, family *Lamiaceae*, is used in Ayurvedic medicine. It shows antioxidant, antidiabetic, antiulcer, and antifungal properties [216,217]. Holy basil decreased mortality in mice after gamma radiation and increased bone marrow stem cell survival [218,219]. Two flavonoids, Ot and Vc, extracted from *Ocimum sanctum* were injected intraperitoneally (50 mg/kg) into mice and irradiated, reducing the number of chromosomal aberrations in bone marrow cells. Neither Ot nor Vc showed toxicity up to the concentration of 200 mg/kg [83]. Polysaccharides of *Ocimum sanctum* have antioxidant properties; they decrease lipid peroxidation and protect plasmid DNA. Moreover, they safeguard mouse splenocytes from death [220].

### 7.9. Emblica officinalis

*Emblica officinalis*, a plant in the family *Euphorbiaceae*, is also known as Amla. The plant is used in Ayurveda; there have been reported antioxidative, anti-inflammatory, antimicrobial, antimutagenic, radiomodulatory, and chemomodulatory properties [221,222]. The plant extract or fruit extract administered in mice prior to gamma radiation resulted in an increase in the survival of animals, a decrease in lipid peroxidation, and a rise in GSH levels in the liver, blood, and brain [223,224]. Moreover, the plant extract normalized the activity of serum alkaline phosphatase. The fruit pulp of Amla also increased the survival of mice after gamma irradiation and countered the body weight loss of the animals [225].

### 7.10. Spinacia oleracea

*Spinacia oleracea*, family *Chenopodiaceae*, a plant growing native in south-west Asia, is used in Indian Ayurveda as a laxative, carminative, in asthma, and in leprosy [226,227]. *S. oleracea* extract protected the mice’s liver against gamma radiation by decreasing lipid peroxidation and increasing the GSH concentration [228]. In another study, the radioprotective potential of *Spinacia oleracea* was tested. The administration of 1100 mg/kg spinach extract to mice 15 days before gamma irradiation decreased lipid peroxidation in the testis, according to its antioxidant activity. Moreover, it also modulates protein, cholesterol, and glycogen values [229].

### 7.11. Panax ginseng

*Panax ginseng*, belonging to the *Araliaceae* family, shows various properties like reducing blood glucose, reducing inflammation, and enhancing vitality [230,231]. The root extract of *P. ginseng* prolonged the lifespan of irradiated mice and countered body weight loss of the animals and damage to the germ cells [232]. Administration prior to irradiation caused a decline in acid phosphatase and lipid peroxidation in mice testes [108]. In another study, the plant showed radioprotective properties to jejunal crypts of mice and inhibited apoptosis [233]. The intake of hydro-alcoholic extract of *Panax ginseng* by Swiss albino mice five days before gamma radiation resulted in increased survival of the animals, and it counteracted weight loss.

Furthermore, it influenced oxidative stress markers like SOD, CAT, and GST; it also reduced lipid peroxidation in the blood and liver [234]. *Panax ginseng* root extract was dispensed to mice prior to 6 Gy gamma irradiation. After only radiation, parameters like erythrocyte, haematocrit values, and haemoglobin concentration were decreased, and there was also an increase in lipid peroxidation in the blood and liver. Pretreatment with the extract alleviated all these parameters; moreover, the GSH level in serum and in the liver was increased [235].

### 7.12. Moringa oleifera

*Moringa oleifera*, family *Moringaceae*, originally comes from India. The plant exhibits antioxidant, antidiabetic, anticancer, antiulcer, and neuroprotective properties [236,237]. M. oleifera leaf methanolic extract injections protected mice bone marrow against post-radiative damage. Due to the administration of the extract, after gamma beams, there was a decrease in chromosomal aberrations in the bone marrow cells of mice. *M. oleifera* leaf extract protected mice liver cells against radiation-induced lipid peroxidation and increased the concentration of reduced GSH [238]. According to the Sinha et al. [239] study, pretreatment of mice with *M. oleifera* leaf extract before irradiation resulted in increases in the activity of antioxidant enzymes like SOD, CAT, and GSH, and in the decline in lipid peroxidation and displacement of NF-κB from cytoplasm in the mice’s liver.

Pradana et al. [240] demonstrated that *Moringa oleifera* extract induced the generation of ROS in epidermoid carcinoma KB cells, suggesting its potential as a radioprotective agent. Additionally, Wang et al. [241] highlighted the antimicrobial activity of *Moringa oleifera*, which could contribute to its radioprotective effects by preventing secondary infections post radiation exposure.

### 7.13. Mesua ferrera

*Mesua ferrera*, family *Clusiaceae*, also named Nagakesara, is used in Ayurveda, Unani and Siddha. The plant has hepatoprotective, antispasmodic, analgesic, antimicrobial, and antiulcer properties [242]. Studies have shown that extracts of Mesua ferrea exhibit antioxidant activity through various assays like DPPH radical-scavenging and ferric reducing power assays, along with anti-inflammatory effects demonstrated by proteinase inhibitor and albumin denaturation assays [243]. Furthermore, *Mesua ferrea* has been found to enhance the activity of antioxidant enzymes, reduce MDA levels, and protect organs such as the liver, kidney, and spleen from gamma radiation-induced damage [244].

### 7.14. Spatholobus suberectus

*Spatholobus suberectus*, family *Fabaceae*, presents wound healing, antioxidative, antiplatelet, antiapoptotic, and anti-inflammatory properties [245,246]. The post-radiation intake of ethanol extract from the plant ameliorated the number of red blood cells, white blood cells, platelets, hemoglobin, and bone marrow cells. The expression of protein Bcl-2 in bone marrow was increased; therefore, apoptosis was inhibited. The levels of antioxidative enzymes were augmented, and the increase in malondialdehyde concentration was countered [247].

### 7.15. Camelia sinensis

*Camelia sinensis* extract is rich in polyphenols, has antioxidative properties, scavenges free radicals, and inhibits lipid peroxidation. The water extract counteracted 100% of radiation-induced strand breaks in pBR322 plasmid DNA at an 80 μg/mL concentration. Moreover, in the study on the V79 cell line, it reduced the number of micronucleated cells and the amount of ROS; it also returned the integrity to the potential of the mitochondrial membrane [248]. Another study showed the influence of the pretreatment of white blood cells and erythroleukemic cells with black tea extract on radiation-induced damage. The extract increased cell viability, reduced the level of ROS and apoptosis in normal cells compared to erythroleukemic cells, and downregulated caspase-3 [249].

Furthermore, fermented black tea shows antioxidant activity according to DPPH radical scavenging. It protects DNA from radiation damage [250]. In another study, tea extract protected radiated skin; the extract inhibited proteasome function modulated NF-κB activity [251].

### 7.16. Nigella sativa

Macerated extract of *Nigella sativa* seeds was administered to mice before whole-body irradiation. The extract showed radioprotection to the liver, brain, spleen, and intestines in normal and tumor-bearing mice according to oxidative stress reduction [252]. Water-based *Nigella sativa* seeds extract reduced malondialdehyde amounts and elevated the activity of antioxidative enzymes like GSHpx and SOD in intestine samples of gamma-irradiated mice. Moreover, it counteracted the radiation-induced damage of jejunal mucosa, for instance, denuded villi and Lieberkühn crypts, ulceration, and congestion in atrophic mucosa [253]. In another study, DPPH free radicals were scavenged by ethanolic extract of *Nigella sativa*. Oral administration of the extract to Swiss albino mice prior to 2 Gy whole-body radiation reduced lipid peroxidation and increased activity of antioxidant enzymes in the spleen and liver.

Furthermore, it prolonged the lifespan of the animals. Extract of *Nigella sativa* also prevented DNA from radiation-induced DNA damage [252]. The study by Çanakci et al. [254] investigates the radioprotective effects of *Nigella sativa* on acute radiation-induced nasal mucositis in rats. The research involved the topical application of black seed oil *from Nigella sativa* to assess its impact on nasal mucosa following radiotherapy. The findings of the study suggest that the topical application of black seed oil demonstrates potential radioprotective effects, as evidenced by the reduction in “superficial erosion” compared to the group treated with saline after irradiation. A recent placebo-controlled study indicates that thymoquinone, the main active ingredient of *Nigella sativa*, demonstrates a radioprotective effect on the lung tissue of rats exposed to IR [255].

## 8. Clinical Studies

There is a limited amount of clinical research examining the effectiveness of natural radioprotectors. There is a need for these to be thoroughly investigated. Clinical studies on vitexin and curcumin have confirmed their effectiveness in a randomized, placebo-controlled comparative clinical trial [256,257]. The efficacy of these substances has also been confirmed in preclinical studies conducted on lymphocytes and blood samples. However, more clinical trials are needed to further confirm their effectiveness. The details of the studies are shown in Table 3.

## 9. Methods of Extraction

Extraction methods are important in the efficiency of obtaining plant extracts rich in bioactive compounds. The type of solvent used significantly influences the extraction efficiency and selectivity of bioactive compounds. Research has shown that the choice of solvent can impact the polarity of the extracted compounds, thereby affecting the overall effectiveness of the extraction process [260]. Various techniques such as ultrasound-assisted extraction (UAE), microwave-assisted extraction (MAE), and supercritical fluid extraction have been extensively researched for their effectiveness in extracting compounds like polyphenols, flavonoids, and essential oils from plant materials [261,262]. These modern extraction methods offer advantages such as higher extraction efficiency, reduced extraction times, and lower energy consumption compared to conventional methods [72]. For example, UAE has been demonstrated to enhance extraction efficiency by inducing acoustic cavitation and facilitating solvent flow into plant cells [263].

The selection of the extraction method can significantly influence the quality and quantity of the extracted compounds. Studies have emphasized the importance of factors such as plant material preparation, solvent selection, extraction technique, and process conditions in determining the extract’s composition and bioactivity [264]. Optimization of extraction conditions, often conducted using response surface methodology (RSM), enables the identification of the most efficient parameters for extracting specific compounds from plants [265,266].

However, it should be noted that the selection of a method should also consider the safety of the solvent used. Some solvents, such as carbon dioxide, are non-toxic, whereas residues of organic solvents must be strictly controlled [267,268].

## 10. Toxicity, Efficacy, and Cost-Effectiveness of Radioprotectors

While natural radioprotectors are generally considered to have lower toxicity profiles compared to synthetic compounds, some challenges remain. Synthetic thiol-containing compounds, although effective as radioprotectors, are limited by their toxicity, including side effects such as hypotension, nausea, and vomiting [269]. Similarly, chemical radioprotectors like AET, WR 2721, and WR 1065 have shown toxic side effects that restrict their clinical utility [161]. The toxicity of these compounds at protective doses hinders their widespread use in medical practice.

However, when using raw plant materials, one should keep in mind their toxicity. A study by Paik et al. [270] showed in a review of cases that high doses or prolonged use of ginseng *(Panax ginseng)* can lead to adverse effects such as insomnia, headaches, digestive issues, and more severe effects like changes in blood pressure and heart rate. Doubts may be raised about genotoxic ginseng. The Kim et al. [271] study evaluated the effect of red ginseng on toxicity, health-related quality of life, and survival after complementary chemotherapy in patients with epithelial ovarian cancer. Red ginseng significantly increased serum alanine aminotransferase and aspartate aminotransferase levels, but they were within normal limits. Moreover, there were no differences in adverse events between the placebo and red ginseng groups. In terms of quality of life, red ginseng was associated with improved emotional functioning and reduced symptoms of fatigue, nausea and vomiting, and shortness of breath, as well as reduced anxiety and disruptions affecting life and improved daytime sleepiness.

*Aloe vera* may have radioprotective benefits but high concentrations or extended use can lead to genotoxic effects, suggesting the need for careful consideration of dosage and duration. Aloe vera is generally considered safe when used as a food flavoring, and the polysaccharide material from the inner gel has been found to be noncytotoxic by the Cosmetic Ingredient Review Expert Panel. However, due to the cytotoxic, mutagenic, and carcinogenic properties of anthraquinones, it is important to monitor the levels of these phenolic compounds in *Aloe vera* whole leaf extract and latex [272,273]. There are recommendations that the maximum allowable aloin content in Aloe-derived materials for oral consumption should be less than 10 parts per million, while for nonmedical use, the recommended limit is 50 ppm or lower [272].

Curcumin, the active component of turmeric, is celebrated for its antioxidant and anti-inflammatory properties and shows potential as a radioprotector. However, excessive consumption or long-term use of curcumin supplements has been linked to liver toxicity. Reports have highlighted cases where high doses of curcumin led to liver function abnormalities and, in some instances, liver damage [274,275].

Coumarin is used as a radioprotector, but its hepatotoxicity necessitates caution. Most patients tolerate coumarin well, though a small group experiences mild to moderate liver toxicity. Instances of hepatotoxicity have led to restrictions or bans on coumarin use in several countries. Further research is needed on gene polymorphisms and environmental factors affecting coumarin metabolism [276].

The toxicity of plant raw materials should be considered individually for each resource. Ensuring the source of raw materials is crucial to avoid contaminants that can affect their safety. Contaminants can come from soil, water, or chemicals used in cultivation. Therefore, it is important to check the origin and quality of plant raw materials before use. Using raw materials from certified organic farms minimizes the risk of exposure to harmful substances.

The efficacy of natural radioprotectors is attributed to their various beneficial activities, which were exhaustively described in this review. Synthetic radioprotectors have also proven effective in safeguarding healthy tissues from radiation damage [42]. Initially, radioprotective compounds were primarily thiol-based synthetics. Amifostine (WR-2721) underwent clinical trials as a viable radioprotector. Amifostine has been FDA-approved for dryness in the mouth caused by radiation therapy [277,278].

The effectiveness of natural and synthetic radioprotectors might vary. The survival rate of cells after 6 Gy radiation treated with crocin is comparable to that of cells treated with amifostine [279]. In another study, amifostine was compared with natural agents like red ginseng, evaluating their efficacy in mitigating the adverse effects of ionizing radiation on various biological parameters, including oxidative stress markers, tissue damage, and cellular apoptosis [280]. The results indicated that both amifostine and red ginseng showed significant reductions in markers of oxidative stress and apoptosis, as well as GSH levels, compared to the control group exposed to radiation alone, suggesting that natural agents like red ginseng may offer comparable protective effects to amifostine against radiation-induced damage, potentially offering a safer and more accessible alternative in clinical settings.

Clinical trials were also conducted to explore the efficacy of both synthetic and natural radioprotectors in combating the side effects of radiotherapy, such as xerostomia. Lee et al. investigated amifostine’s effectiveness in reducing severe xerostomia during and after radiotherapy for head-and-neck squamous-cell carcinoma. However, it failed to significantly decrease grade ≥2 acute or late xerostomia compared to placebo, and it also showed increased adverse effects [281]. In contrast, Ameri et al. investigated the efficacy of an herbal compound containing *Malva sylvestris* and *Alcea digitata* vs. artificial saliva for alleviating symptoms of at least grade 1 radiation-induced xerostomia post radiotherapy [282]. Both interventions improved symptoms, but the herbal compound demonstrated additional benefits in improving the severity of dry mouth. The third study evaluated the use of 1% pilocarpine mouthwash versus placebo in patients with radiation-induced xerostomia [283]. It was found that pilocarpine mouthwash effectively maintained saliva production after radiotherapy compared to placebo. While amifostine exhibited limited efficacy and more side effects, both the herbal compound and pilocarpine mouthwash emerged as promising treatments for managing radiation-induced xerostomia in head-and-neck cancer patients.

The efficacy of herbal extracts hinges significantly on their active constituents, whose concentration can vary due to factors such as the method of extraction, climatic conditions, geographic origin, and the timing of sample collection [284,285,286]. These variables contribute to inconsistencies in the overall effectiveness of herbal preparations. However, ensuring proper characterization and rigorous scientific evaluation of herbal products can lead to the development of an ideal radioprotector with minimal toxicity and maximal efficacy. Standardization becomes crucial in this context, as it allows for consistency in the composition and potency of herbal extracts across different batches [287,288]. This ensures repeatability in outcomes and guarantees safety and efficacy in clinical applications. By establishing standardized protocols for the preparation, quality control, and assessment of herbal extracts, researchers can enhance reliability in their therapeutic effects and pave the way for safer and more effective treatments in radioprotection and beyond [289].

When considering radioprotectors for cancer patients, it is crucial to factor in both the costs of natural and synthetic options, as affordability plays a significant role in treatment decisions. Natural radioprotectors, sourced from botanicals and plant extracts, are often perceived as cost-effective due to their potentially lower production costs and easy availability. In contrast, synthetic radioprotectors like amifostine may involve higher production expenses for patients. The economic evaluation of synthetic radioprotectors should consider manufacturing costs and administration expenses. In a 2006 study on non-small-cell lung cancer patients, using amifostine incurred an average cost of USD 4421 per patient compared to USD 2709 for those not receiving it, reflecting an incremental cost of USD 1711 [290]. Currently, based on data from the drugs.com website, the cost of Ethyol^®^ (amifostine intravenous powder for injection 500 mg) is around USD 481 for a supply of 1 g powder for injection [291].

## 11. Natural Radioprotectants Patents

Natural radioprotectants show significant potential due to their ability to mitigate the harmful effects of radiation exposure. Table 4 displays patented solutions involving various natural radioprotectants, solutions containing natural compounds or plant materials, or their preparations.

## 12. Conclusions

The studies presented suggest that plant-derived substances and extracts show promise in therapy. They have various beneficial effects that could help reduce the adverse effects of radiation therapy through different mechanisms.

The intricate interactions and biological effects exerted by these natural compounds require a thorough understanding. Future research should concentrate on comprehensively grasping the mechanisms through which these substances can protect healthy cells and support anticancer therapy. This approach will enable us to optimize therapeutic strategies and customize treatment for individual patients. The diversity of natural compounds poses challenges in understanding, yet it also presents an opportunity to discover effective therapies with potential benefits for anticancer treatment and radioprotective effect. However, there are challenges in analyzing these extracts, ensuring their origin and quality, and reproducing them consistently across different varieties. It is challenging to analyze extracts and guarantee their origin and quality accurately. Without proper controls and standardized methods, it is hard to use these substances in a controlled way. To address these challenges, we need better strategies for analyzing these extracts, ensuring quality, and ensuring they are consistent across different types of plants. Collaboration between researchers, doctors, and regulators could help establish guidelines for using these plant-derived compounds in therapy. By overcoming these challenges, we can fully utilize the potential of plant-derived substances to improve existing cancer treatments. It would provide patients with more effective and safer options for managing their condition.

## Figures and Tables

**Table 1:** In vitro studies on radiomodulating effects of natural compounds.

Cell Line	Radioprotective Agents Studied	Methods/Assays Used	Type of Radiation	Results	Authors
Chinese hamster ovary (CHO) cells	Selenium Nanoparticles in Aqueous Rosemary Extract, Rosemary extract	MTT assay	X-ray	Similar radiation protection effects were observed for nanoparticles and rosemary.	Hasanzadeh et al., 2022 [67]
Human peripheral blood lymphocytes	Herniarin	Micronucleus assay, Flow cytometry, ROS level analysis	X-ray	Herniarin reduced radiation-induced cytotoxicity and genotoxicity.	Al Fares et al., 2022 [68]
Normal human lung cells (HEL 299 Cells)	Delphinidin	MTT assay, 2′-7′-dicholordihydrofluorescein diacetate assay, SOD activity assay, CAT activity assay, Western blot assay (DNA damage-induced cellular apoptosis)	Proton Beam	Delphinidin showed radioprotective effects, including restoration of antioxidant enzyme activities, increased pro-survival protein levels, and decreased pro-apoptosis protein levels.	Kim et al., 2018 [69]
Human keratinocyte (HaCaT)	Interruptin C from *Cyclosorus terminans*	SOD activity assay, Clonogenic cell survival, Micronuclei formation assays (DNA damage and cell cycle progression), γH2AX assay (DNA repair after irradiation), Western blotting (the levels of the proteins related to the radioprotective responses)	X-ray	Interruptin C increased antioxidant activity, decreased DNA damage, decreased apoptotic protein levels, increased antiapoptotic protein levels, and increased cell survival following irradiation.	Chumsuwan et al., 2022 [70]
Human cervical cancer cells (HeLa)	*Haberlea rhodopensis* Extract	Redox components assessment (lipid peroxidation test, total GSH levels, CAT enzyme activity, SOD enzyme assay, glutathione peroxidase (GSHpx) activity), Comet assay, Flow cytometry (cell cycle), Gene transcription assessment using RT-qPCR	γ-rays	*Haberlea rhodopensis* extract reduced the severity of genotoxic and oxidative stress in HeLa cells.	Staneva et al., 2023 [71]
	Sinomenine hydrochloride from *Sinomenium acutum*	MTT assay, Colony forming assay, Apoptosis and cell cycle assay, DNA repair capacity (Immunofluorescence), Comet assay	X-ray	SH enhanced HeLa cell sensitivity to IR by increasing DNA double-strand breaks and disrupting DNA damage checkpoint activation.	Zhang et al., 2018 [72]
Normal breast cells (MCF-10A)	Interruptin C from *Cyclosorus terminans*	SOD activity assay, Clonogenic cell survival, Micronuclei formation assays (DNA damage and cell cycle progression), γH2AX assay (DNA repair after irradiation), Western blotting (the levels of the proteins related to the radioprotective responses)	X-ray	Interruptin C increased antioxidant activity, decreased DNA damage, decreased apoptotic protein levels, increased antiapoptotic protein levels, and increased cell survival following irradiation.	Chumsuwan et al., 2022 [70]
Human breast cancer cell lines (MDA-MB-231 and Hs578T)	Interruptin C from *Cyclosorus terminans*	SOD activity assay, Clonogenic cell survival, Micronuclei formation assays (DNA damage and cell cycle progression), γH2AX assay (DNA repair after irradiation), Western blotting (the levels of the proteins related to the radioprotective responses)	X-ray	Interruptin C did not promote cell survival.	Chumsuwan et al., 2022 [70]
Adenocarcinomic human alveolar basal epithelial cells (A549)	Isorhamnetin	MTT assay, Colony formation assay, Micronucleus assay, Immunostaining, Apoptosis assays, Level of cytokines measurement via meso scale discovery assay, Mitochondrial membrane potential measurement, RNA interference, Cell growth curve	X-ray	Isorhamnetin caused radio sensitization, decreased colony formation, increased DNA damage, enhanced apoptosis, suppressed NF-κB signaling, and upregulated IL-13.	Du et al., 2021 [73]
Human prostate cancer cell line (PC-3)	Auraptene	Cell viability assay, Apoptosis detection (flow cytometry), Gene expression evaluation	X-ray	Auraptene as a radiosensitizer decreased cell viability and increased apoptosis, reduced survival fraction, induced P53 and BAX expression, and downregulated expression of BCL2, GATA6, and CCND1.	Abolhassani et al., 2023 [74]
Human colorectal adenocarcinoma (HT-29)	Thymoquinone	MTT cell proliferation assay, Clonogenic survival assay, Cell cycle analysis, Sphere formation assay	X-ray	Thymoquinone as a radiosensitizer reduced cell viability and clonogenic survival, and inhibited sphere formation.	Al Bitar et al., 2022 [75]
Human breast cancer (MDA-MB-231)	Dalbergin	MTT assay, Clonogenic survival assay, The gene expression level	X-ray	Dalbergin acted as a radiosensitizer; it inhibited cell proliferation and showed apoptotic effects, probably through the STAT/p53 signaling pathway.	Valojerdi et al., 2023 [76]
	*Anagallis arvensis* extract	Cell cycle arrest, Apoptosis assay, Gene expression	γ-rays	The extract acted as a radiosensitizer; it reduced cell cycle progression and cell growth via induced apoptosis.	Hassan et al., 2022 [77]
Human breast cancer (T47D)	Dalbergin	MTT assay, Clonogenic survival assay, The gene expression level	X-ray	Dalbergin acted as a radiosensitizer; it suppressed cell proliferation and induced apoptosis, likely mediated by the STAT/p53 signaling pathway.	Valojerdi et al., 2023 [76]
Hepatocellular carcinoma cell line (HepG2)	Pomegranate peel extract	MTT assay, Proliferation and apoptotic parameters, Apoptosis assay, Defensive effects against oxidative and antioxidant status	γ-rays	The extract acted as a radiosensitizer, slowed the proliferation of cancer cells, enhanced apoptosis (induction of tumor PPAR-γ and caspase-3), increased Nrf-2, SOD, and CAT activities, and decreased MDA concentration.	Elbakry et al., 2023 [78]
Human breast cancer cells (MCF-7)	*Anagallis arvensis* extract	Cell cycle arrest, Apoptosis assay, The gene expression	γ-rays	The extract acted as a radiosensitizer; it reduced cell cycle progression and cell growth via induced apoptosis.	Hassan et al., 2022 [77]
Human glioblastoma (U251)	Gallic acid gold nanoparticles	MTT assay, Cell cycle and cell death analysis, Western blotting	X-ray	Nanoparticles inhibited cell survival, increased radiation-induced cell death, and arrested the cell cycle.	Jing et al., 2021 [79]
Human umbilical vein endothelial cells	*Olea europaea* L. cv. Caiazzana Leaf extract	β-Galactosidase assay, Radiation-induced DNA damage assay	X-ray	Reduction in the frequency of radiation-induced micronucleus formation and the onset of premature senescence was delayed.	Pacifico et al., 2022 [80]
Primary prostate adenocarcinoma (DU145)	*Olea europaea* L. cv. Caiazzana Leaf extract	β-Galactosidase assay, Radiation-induced DNA damage assay	X-ray	The extract acted as a radiosensitizer; genotoxicity was increased.	Pacifico et al., 2022 [80]
Non-transformed human mammary epithelial (MCF-10A) cells	*Olea europaea* L. cv. Caiazzana Leaf extract	β-Galactosidase assay, Radiation-induced DNA damage assay	X-ray	Reduction in the frequency of radiation-induced micronucleus formation.	Pacifico et al., 2022 [80]
Human pancreatic epithelioid carcinoma (PANC-1) cells	*Olea europaea* L. cv. Caiazzana Leaf extract	β-Galactosidase assay, Radiation-induced DNA damage assay	X-ray	The extract acted as a radiosensitizer; genotoxicity was increased.	Pacifico et al., 2022 [80]
Human lymphoma (U937) cells	Withaferin A	Cellular viability assay, Analysis of mitochondrial transmembrane potential, Measurement of ROS	X-ray	Withaferin A increased apoptosis as a radiosensitizer.	Yang et al., 2011 [81]

**Table 3:** Clinical and preclinical studies on the radioprotective efficacy of substances of natural origin.

Type of Study	Model	Dosage Method Used	Applied Radiation	Results	Authors
Clinical studies					
Randomized, placebo-controlled comparative clinical trial	72 participants	Oral 400 mg of vitexin daily 6 weeks	Radiation therapy; total dose of radiation (Cobalt-60) was 5000 rad	Improvement in the overall health of breast cancer patients undergoing treatment, with restoration of peripheral blood cells, as well as lymphocyte function.	Van Hien et al. [257]
Randomized double-blinded placebo-controlled clinical trial	45 participants	Oral 3 g of curcumin, daily 1 week before the initiation of radiotherapy until its completion	Radiation therapy; total dose of 74 Gy	Curcumin may increase total antioxidant capacity (TAC) while decreasing the activity of antioxidant enzymes such as superoxide dismutase (SOD) in patients with prostate cancer undergoing radiation therapy. In these patients, curcumin improves the antioxidant status without affecting the therapeutic efficacy of radiotherapy.	Hejezi et al. [256]
Preclinical studies					
Preclinical	Human lymphocytes obtained from healthy individuals	Standardized North American ginseng extract (NAGE, total ginsenoside content: 11.7%) 250–1000 microg mL^−1^ at 90 min post irradiation	Ex vivo irradiation 1 and 2 Gy (0.6 Gy/min) (Gamma Cell 40, Radiation Machinery, Ontario, Canada)	A NAGE concentration of 750 μg/mL reduced the incidence of micronucleation by 50.7% after exposure to a dose of 1 Gy and by 35.9% after exposure to a dose of 2 Gy (comparable to those obtained with WR-1065); NAGE was found to reduce the incidence of micronucleation and the level of reactive oxygen species (ROS), while increasing the total antioxidant capacity (TAC) in lymphocytes.	Lee et al. [258]
Preclinical	Peripheral blood samples after a single oral ingestion of 500 mg hawthorn powder extract	500 mg hawthorn powder extract 10 min before and 1, 2, and 3 h after ingestion	150 cGy cobalt-60 gamma irradiation	The maximum decrease in the frequency of cells containing micronuclei was observed 1 h after ingestion of hawthorn extract (an average decrease of 44%).	Hosseinimehr et al. [259]

**Table 4:** Patented solutions of natural radioprotectants.

Title of Patent	Application Date	Subject of the Patent	Country	Number
Methods of using beta glucan as a radioprotective agent	2008-07-08	The invention involves using β(1,3; 1,6) glucan to treat and prevent radiation or chemotherapy-related injuries.	United States	US8563531B2
Anti-inflammatory quinic acid derivatives for radioprotection/radiomitigation	2010-05-11	Methods using analogs of quinic or shikimic acids protect from ionizing radiation effects, pre or post exposure, useful in treating humans and animals at risk for radiation sickness/death.	United States	WO2010132504A1
Antiradiation black tea composition and preparation method thereof	2015-11-16	The invention introduces a radioprotective milk vinegar–green tea beverage with cattle milk, green tea, and Chinese herbs.	China	CN105770514A
Skin radiation-preventing composition	2011-11-14	Skin radiation-preventing composition with vitamins, herbs, and natural extracts enhances skin’s resistance to ionizing radiation effectively.	China	CN102362846A
Radioprotection agent	2006-03-21	Mixture of ecdysteroids from crown saw-wort acts as radioprotective agent, reducing genotoxic effects of gamma radiation.	Russia	RU2326672C2
Composition comprising *Polyopes lancifolia* (harvey) kawaguchi et wang extract for protecting against radiation	2011-01-03	Composition with *Polyopes lancifolia* extract protects cells or tissues against gamma radiation damage, enhancing organismal resistance.	South Korea	KR20120078863A
Composition for immune boosting having radioprotective effect comprising fucoidane from *Ecklonia cava* extracts	2011-07-25	The composition, containing *Ecklonia cava*-derived fucoidan, enhances immunity and provides protection against ionizing radiation, thereby supporting overall health. The fucoidan is extracted using saccharolytic enzymes and proteases.	South Korea	KR20130012417A
Traditional Chinese medicine composition cooperatively used in radiotherapy	2013-11-03	The invention is a traditional Chinese medicine composition containing various herbs for cooperative use in radiotherapy to reduce side effects and enhance cancer cell sensitivity.	China	CN103550506A
Antiradiation injury medicine	2005-12-15	The invention is a medicament made from tricholoma matsutake polysaccharide and ginseng, preventing and treating radiation-induced injuries, such as free radical damage, immune system impairment, hematopoietic damage, and tumor cell growth.	China	CN100360137C
Oil palm phenolics composition for the protection of humans, organs, cells, and tissues against the injurious effects of exposure to ionizing radiation	2016-12-30	The invention is a composition comprising oil palm phenolics or vegetation liquor extract obtained from the aqueous stream of palm oil milling effluent, designed to mitigate the effects of ionizing radiation.	Malaysia	WO2017116225A1
Compound for preventing and treating radiation damage and the preparing method	2007-04-29	The invention is a method using curcuma longa and cape jasmine to prevent ionizing radiation damage.	China	CN101041070A
Nursing medicine for skin injury after radiotherapy	2014-04-10	The nursing medicine for skin injury after radiotherapy is prepared from the following herbs in percentage by weight: auriculate swallowwort root, bulbophyllum herb, ivy glorybind herb root, herba siegesbeckiae, etc.	China	CN103877387A
Compound Chinese medicine preparation for assisting tumor radiotherapy and its preparation method	2007-02-01	The compound Chinese medicine preparation for assisting tumor radiotherapy includes rehmannia root, astragalus root, angelica, figwort, ophiopogon root, etc.	China	CN101020002B
Plant pulp in preparation of medicines and health foods for treating injury of ovary caused by radiotherapy and chemotherapy	2020-06-10	The invention focuses on utilizing sea buckthorn pulp in the preparation of medicines and health foods specifically aimed at treating ovary injury caused by radiotherapy and chemotherapy.	China	CN111643532A
Snake medicine oil for radiotherapy protection and preparation method thereof	2022-12-06	The invention introduces snake medicine oil for protecting against radiotherapy and provides its preparation method. The method involves weighing and mixing specific medicinal materials including paeonia ostii, borneol, mint, frankincense, houttuynia cordata, angelica sinensis, honeysuckle, gardenia, astragalus membranaceus, etc.	China	CN115779021A
Application of nano-selenium *Cordyceps militaris* aqueous extract for reduction in radiotherapy injury and protective agent thereof	2021-11-13	The invention pertains to an extract of nano-selenium cordyceps militaris used for reducing radiotherapy injury, particularly mitigating damage to organs or muscles and decreasing ROS elevation caused by radiotherapy.	China	CN113908180A
Medical ray protection spray containing plant exosome	2020-09-29	The invention is a medical ray protection spray containing plant exosomes, superoxide dismutase, rose exosome freeze-dried powder, and purified water, designed to protect against medical radiation.	China	CN112220914A
Medical ray protection spray and use method thereof	2022-04-20	The invention discloses a medical ray protection spray and its use method in the field of medicine. The spray includes superoxide dismutase, vitamin B12, potassium sorbate, *radix Angelicae pubescentis*, sorbitol, aloe, chamomile, and purified water	China	CN114832097A
Medical ray protective agent and preparation method thereof	2020-10-26	The invention is a medical ray protective agent containing perilla anthocyanin extract, honeysuckle stem extract, plant essential oil, and curcumin. These plants scavenge free radicals, prevent radioactive skin injury, and reduce radiodermatitis occurrence.	China	CN112206316A
Composition for regeneration and protection of the skin	2016-09-29	The composition for skin regeneration and protection includes rosehip oil, snail slime, *Aloe vera* gel, *Onopordum acanthium* extract, and rockrose essential oil. It is used to prevent or treat skin damage from radiotherapy, surgery, burns, wounds, and for cosmetic treatment of epidermal damage.	Spain	ES2661576A1
Medical antiradiation nursing gel and preparation method thereof	2021-07-15	The invention concerns a medical antiradiation nursing gel and its preparation method. The gel comprises aloe gel, ginkgo biloba extract, allantoin, glycyrrhetinic acid, vitamin E dry powder, hyaluronic acid, saffron crocus extract, grape seed extract, and Arabic gum.	China	CN113425798A
Application of baicalein to preparation of medicines for treating and preventing ionizing radiation injury	2019-09-24	The invention applies baicalein and its pharmaceutically usable salts to the preparation of medicines for treating or preventing ionizing radiation injury.	China	CN110448550A

## Abbreviations

**Abbreviation**	**Component**
ABTS	2,2′-Azino-bis(3-ethylbenzothiazoline-6-sulfonic acid) radical
A549	Adenocarcinomic Human Alveolar Basal Epithelial Cells
ATP	Adenosine 5γ-Triphosphate
CAT	Catalase
CGRP-TNF-α	Calcitonin Gene-Related Peptide-Tumor Necrosis Factor Alpha
CHO	Chinese Hamster Ovary Cell Line
CPK	Creatine Phosphokinase
DPPH	2,2-Diphenyl-1-picrylhydrazyl
FDA	US Food and Drug Administration
GSH	Glutathione
GSHpx	Glutathione Peroxidase
GST	Glutathione S-Transferase
HaCaT	Human Keratinocyte
HEI-OC1	House Ear Institute–Organ of Corti 1
HEL 299	Normal Human Lung Cells
HepG2	Hepatocellular Carcinoma Cell Line
HMGB1	High Mobility Group Box 1
HO-1	Heme Oxygenase-1
HRE	*Haberlea rhodopensis* Extract
HT-29	Human Colorectal Adenocarcinoma Cell Line
IGF-1	Insulin-like Growth Factor 1
IL-1β	Interleukin-1 Beta
IR	Ionizing Radiation
MAPK	Mitogen-Activated Protein Kinase
MCF-10A	Non-transformed Human Mammary Epithelial Cell Line
MCF-7	Human Breast Cancer Cells
MDA	Malondialdehyde
MDA-MB-231	Breast Cancer Cell Line
MPG	2-Mercaptopropionyl Glycine
Nrf2	Nuclear Factor Erythroid 2-Related Factor 2
NK	Natural Killer
NO•	Nitroxyl Anion
Ot	Orientin
PANC-1	Human Pancreatic Epithelioid Carcinoma Cell Line
PC-3	Human Prostate Cancer Cell Line
PPAR-γ	Peroxisome Proliferator-Activated Receptor Gamma
PKB	Protein Kinase B
PKC	Protein Kinase C
RIII	Radiation-Induced Intestinal Injury
RN	Radiation Nephropathy
ROS	Reactive Oxygen Species
SMGs	Submandibular Glands
SOD	Superoxide Dismutase
STAT	Signal Transducer and Activator of Transcription
T47D	Human Breast Cancer Cell Line
TGF-β	Transforming Growth Factor Beta
TNF-α	Tumor Necrosis Factor Alpha
VEGF	Vascular Endothelial Growth Factor
Vc	Vicenin
WHO	World Health Organization
YSE	*Yuca schidigera* Extract
